# High‐Power Terahertz Emission from Picosecond Nano‐Plasma Switching Driven by Secondary Electron Emission Avalanche

**DOI:** 10.1002/advs.75300

**Published:** 2026-04-21

**Authors:** Guangyu Sun, Mohammad Rezaei, Yuheng Hu, Guan‐Jun Zhang, Elison Matioli

**Affiliations:** ^1^ Power and Wide‐band‐gap Electronics Research Laboratory (POWERlab) Institute of Electrical and Micro Engineering École Polytechnique Fédérale de Lausanne (EPFL) Lausanne Switzerland; ^2^ State Key Laboratory of Electrical Insulation and Power Equipment School of Electrical Engineering Xi'an Jiaotong University Xi'an China

**Keywords:** field emission, nano‐plasma, nanoelectronics, secondary electron emission, terahertz

## Abstract

Developing compact, high‐power sources in the terahertz (THz) gap (0.1–10 THz) remains a critical challenge in modern electronics, and nano‐plasma device (NPD) represents a promising pathway to close this gap. Here, we advance the fundamental understanding of NPDs by showing that an ultra‐dense (∼10^25^ m^−3^) electron sheet, confined within ∼10 nm above the substrate surface, drives the picosecond nano‐plasma switching through secondary electron emission avalanche (SEEA) on the substrate surface. This mechanism was validated by analytical theories, particle‐in‐cell simulations, and THz experiments, showing that a minimum electric field of ∼10^8^ V/m is required for SEEA initiation, which covers an ∼100 nm gap within ∼ 0.1 ps, switches the NPD on via ionization avalanche within ∼ 1 ps, and produces a 0.4 THz signal with 2 W power. The switching speed increases with higher pressure. Model‐guided device optimization identified the high‐secondary‐emission‐yield substrates that favor THz generation, and defined the optimum nanogap length range that ensures both discharge initiation and emission within the THz range. This work demonstrates NPD as a feasible and scalable design for watt‐level, on‐chip THz generation, and suggests possible future integration with triboelectric nanogenerators as well as potential implications for vacuum nanoelectronic platforms.

## Graphical abstract

A nano‐plasma device enables watt‐level on‐chip THz generation through picosecond switching triggered by secondary electron emission avalanche. An ultra‐dense electron sheet initiates nano‐plasma formation within a ∼100 nm gap, driving rapid energy release into an integrated resonator. The mechanism reveals a feasible and scalable route toward high‐power, chip‐scale THz sources.

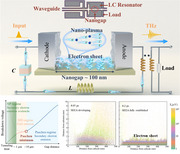

## 1 Introduction

Terahertz (THz) radiation has attracted enormous attention owing to its broad range of applications in spectroscopy [1, 2, 3, 4, 5, 6], sensing [7, 8, 9, 10, 11, 12], biomedicine [13, 14, 15], ultrafast optics [16, 17, 18], and next‐generation wireless communication [19, 20, 21]. These applications demand compact THz sources that can simultaneously deliver high output power, broad bandwidth, and high energy efficiency. Conventional THz emitters are typically classified into solid‐state electronic devices, photonic devices, and vacuum‐electronic devices [22, 23, 24, 25, 26]. Despite substantial progress, solid‐state electronic emitters remain constrained by limited speed and low output power at higher frequencies [27], while photonic emitters often require cryogenic cooling or bulky laser systems. Vacuum‐electronic sources deliver high THz power, but are not compatible with chip‐scale applications [28]. More recently, emerging concepts such as nano‐plasma devices (NPD) [29], nanoscale air channel devices [30, 31], electronic metadevices [32, 33], 2D materials [34, 35, 36], metasurfaces [37, 38], spintronic emitters [39, 40, 41], nanotube [42], and Brillouin oscillators [43] have opened new pathways to THz generation that may overcome the limitations of conventional devices.

Among these concepts, NPDs stand out for their watt‐level output power that is orders of magnitude higher than conventional solid‐state electronic and photonic sources, in addition to an ultrafast switching speed of ∼10 V/ps that is two orders of magnitude faster than state‐of‐the‐art field‐effect transistors [44, 45, 46]. The promising performance of NPDs critically relies on the picosecond nano‐plasma switching, yet this process cannot be captured by existing diagnostics or discharge models. This lack of understanding prevents systematic improvement of essential device metrics such as breakdown voltage, output frequency, reliability, and lifetime. Consequently, current optimization strategies remain largely empirical, such as varying gap length [45], electrode geometry [47], and materials [48], underscoring the need for a comprehensive framework to guide NPD design.

**FIGURE 1:**
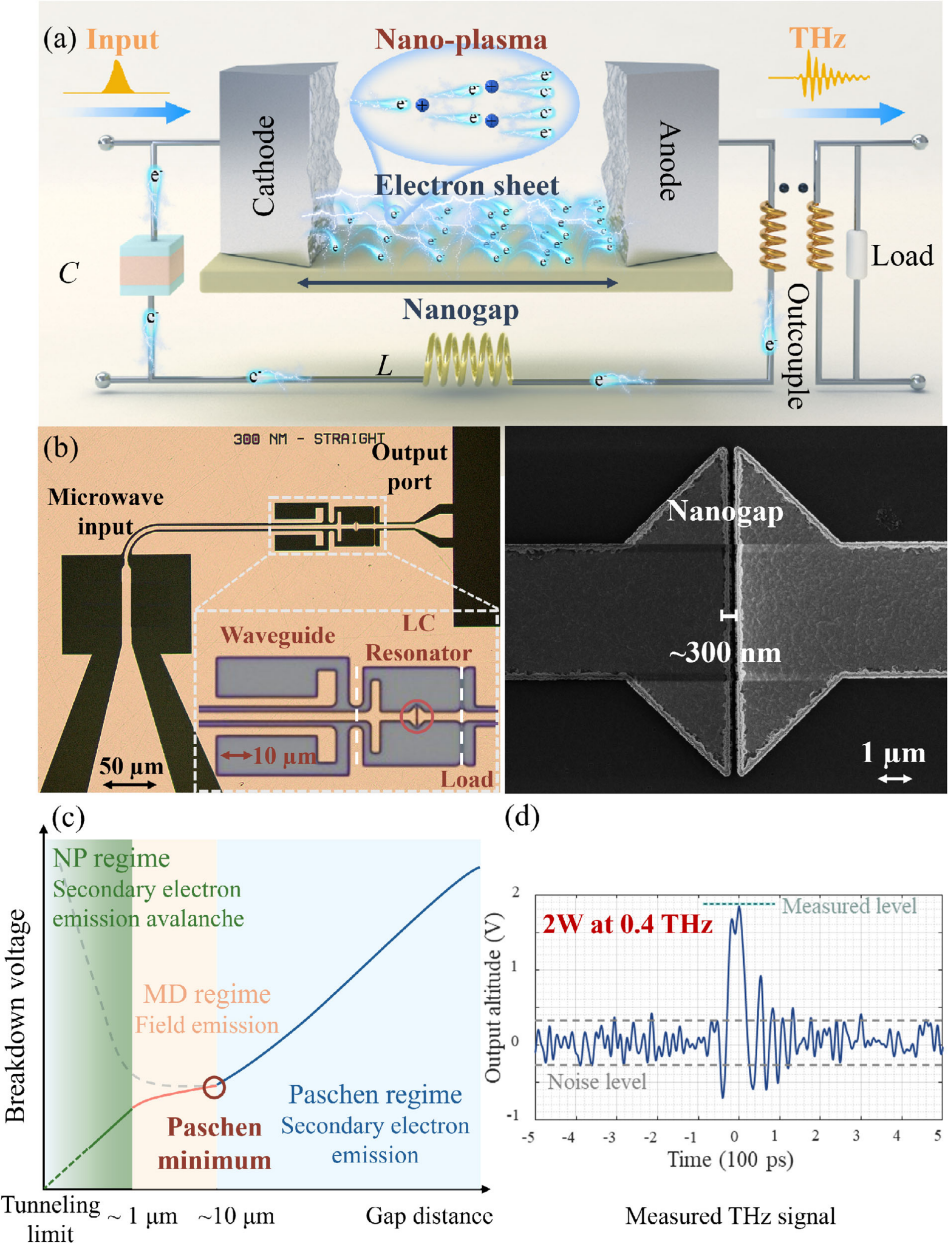
Overview of nano‐plasma device for THz generation. (a) Schematic of picosecond nano‐plasma switching driven by the SEEA, forming an ultra‐dense electron sheet and nano‐plasma. The input signal, output THz signal, and device components are illustrated. (b) SEM image of the system design, including the microwave input, output port, and the NPD composed of a waveguide structure, LC resonator, and nanogap (red‐circled). (c) Breakdown voltage as a function of gap length. Three regimes, including the classic Paschen regime (>∼10 µm), the micro‐discharge regime (∼1–10 µm), and the nano‐plasma regime (<∼1 µm), are indicated. The tunneling limit at ∼1 nm and the Paschen minimum are also shown. (d) Measured down‐converted THz signal generated by the nano‐plasma device. A 0.4 THz signal with a peak power of 2 W was measured.

Existing micro‐discharge models developed for sub‐10‐µm gaps cannot be applied to nano‐plasma [49, 50, 51, 52]. At atmospheric pressure, micro‐discharge is attributed to Townsend‐like ionization avalanche induced by field‐emission (FE) [53, 54], where the breakdown voltage is dictated by the modified Paschen's law and decreases as the gap is reduced (orange region in Figure 1c) [55, 56]. However, the micro‐discharge models predict that no discharge can happen when the gap is below a threshold set by the Townsend breakdown criterion: [52, 57]:

(1) $$d_{\mathrm{gap},\min}^{\mathrm{Townsend}}=\lambda_{\mathrm{ion}}\ln\left(1+\frac{1}{\gamma }\right)$$

where λ_ion_≈1.7 µm [58] is the ionization mean free path in atmospheric air, and γ∼0‐1 [59] is the effective cathode emission coefficient. The typical gap length of NPDs is on the order of 100 nm and is well below $d_{\mathrm{gap},\min}^{\mathrm{Townsend}}$ [29, 44, 47, 48]. Therefore, NPD operation is beyond the scope of conventional micro‐discharge models.

Previous studies of nano‐plasma have largely focused on transient plasmas generated by lasers or X‐rays under vacuum conditions [60, 61, 62, 63, 64], as well as nanotip and nanoprotrusion breakdowns [65, 66, 67, 68]. However, plasma behavior in NPDs is remarkably different and requires more in‐depth investigations, as NPDs operate in atmospheric pressure and are initiated purely by pulsed electric fields to produce repeatable discharges. These features are not unique to NPDs but also appear in a variety of high‐field nanoelectronic devices, such as nanoelectromechanical systems [69], advanced interconnects [70], 2D transistors [70, 71], nanoscale sensors [72, 73], and diodes [74, 75, 76], highlighting the broad relevance of a nanogap air breakdown framework.

In this work, we reveal that picosecond nano‐plasma switching can be driven at atmospheric air by the formation of an ultra‐dense (∼10^25^ m^−3^) electron sheet (UES) confined within ∼10 nm of the substrate via a secondary electron emission avalanche (SEEA) mechanism. The UES is generated by the cathode field emission electrons multiplying via a cascade of secondary electron emissions on the substrate surface. This model was validated through analytical theory, particle‐in‐cell simulations, and experiments, showing excellent consistency across key quantities including the switching time, surface charge density, electron sheet thickness, and charge density. Device optimization based on the proposed framework was performed, identifying substrate materials with high secondary electron yield (SEY) that favor the THz generation, and determining the optimum discharge gap length ensuring both discharge initiation and emission within the THz range. In addition, potential applications of the proposed framework were discussed, including possible SEEA or UES‐inspired concepts for vacuum nanoelectronic devices and the development of triboelectric nano‐plasma systems. These results demonstrate NPDs as a robust and scalable design for on‐chip, high‐power THz generation compatible with established and emerging nanosystems.

## 2 Results and Analyses

### 2.1 The SEEA‐Driven Picosecond Nano‐Plasma Switching

The nano‐plasma device for THz generation is illustrated by the schematic and scanning electron microscope (SEM) image in Figure 1a,b. A nanogap for discharge is formed by depositing metallic electrodes with a thickness of a few hundred nanometers on a substrate, with a gap length ranging from 20 to 500 nm. The nanogap is integrated with an LC resonator, a coplanar waveguide structure, and a load for THz emission. Device fabrication is detailed in Section 5.1 and in our previous works [29, 44].

The THz emission mechanism is briefly explained as follows. Under microwave excitation, the capacitor *C* is gradually charged while the nano‐plasma device stays in the pre‐breakdown insulating state with only a small current. As the applied voltage approaches the threshold breakdown voltage, the current increases sharply, indicating the OFF‐to‐ON switching event (Figure S1), in contrast to the gradual turn‐on behavior of conventional solid‐state devices. When the threshold is reached, nano‐plasma is generated within the picosecond timescale, and the associated ultrafast switching edge gives rise to broadband radiation extending well into the THz range. The LC resonator connected to the NPD then selects and enhances the component at its eigenfrequency $f_{0}=1/\left(2\pi \sqrt{LC}\right)$, leading to a short burst of THz radiation.

The down‐converted THz measurement is shown in Figure 1d, where for a designed *f*_0_ of 0.4 THz, an output power of 2 W was measured, which is orders of magnitude higher than conventional semiconductor THz devices. THz measurement setup and power calculation are described in Section 5.2. Efficient excitation of the THz resonance requires that the switching time *t*_on_ be shorter than the oscillation period of the target eigenfrequency, i.e. $t_{\mathrm{on},\max}∼1/\left(2\pi f_{0}\right)$ (derivations are shown in Equation S1). Therefore, the picosecond switching feature intrinsic to the NPD is essential for the THz generation.

The schematic in Figure 1a illustrates the nano‐plasma generation mechanism via SEEA. Field‐emission electrons multiply via secondary electron emission on the substrate, forming a UES and inducing the ionization avalanche, which is considered impossible in a nanogap according to the classic micro‐discharge model.

**FIGURE 2:**
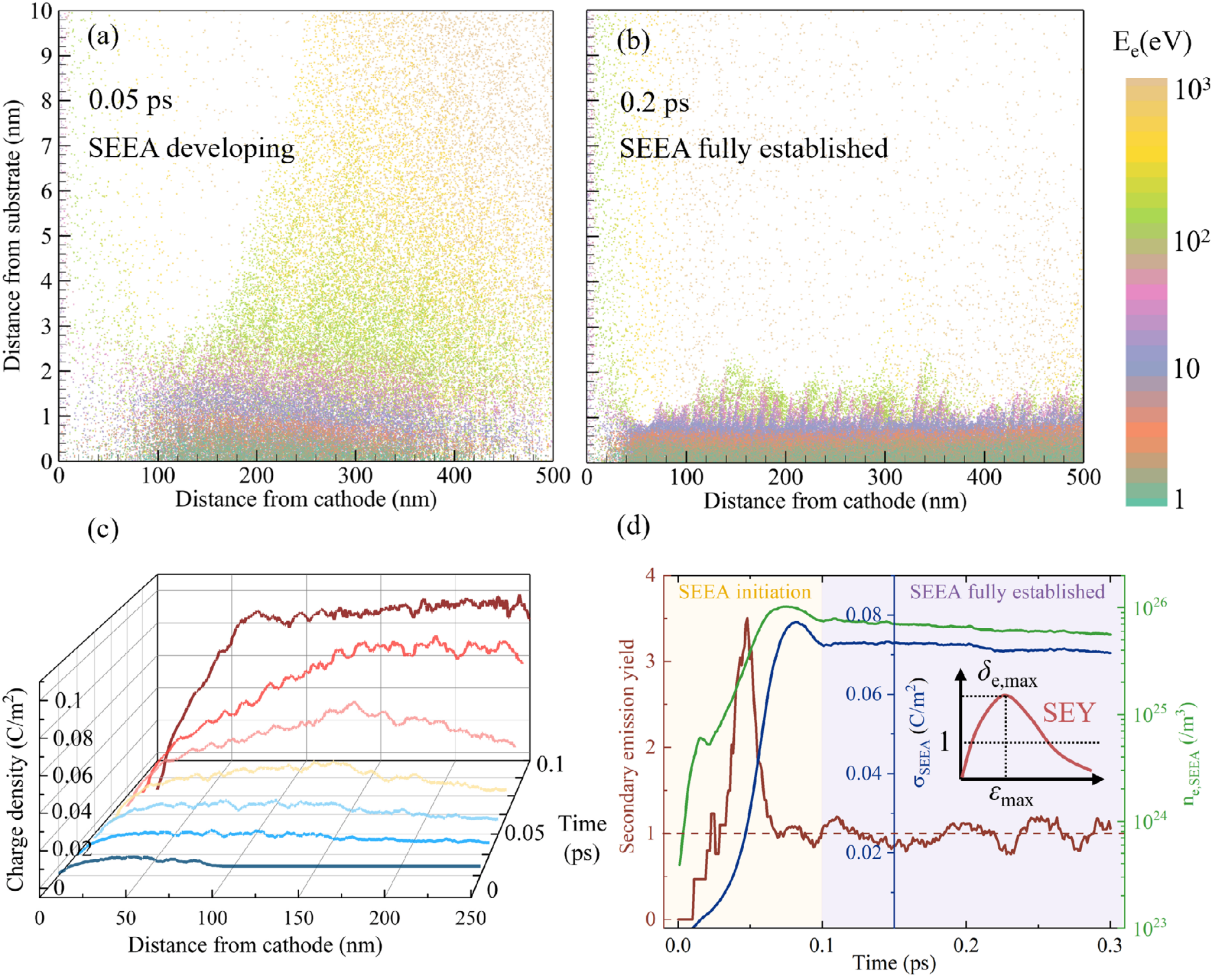
Numerical simulation of the ultra‐dense electron sheet formation during secondary electron emission avalanche. (a, b) Real‐time electron distribution above an NPD with a 500 nm gap at (a) 0.05 ps and (b) 0.2 ps, showing a SEEA fully developed within 0.2 ps. The color scale represents the electron energy. (c)‐(d) SEEA development in an NPD with a 250 nm gap. (b) Evolution of surface charge distribution from 0 to 0.1 ps. (c) Time evolution of SEY, surface charge density, and electron sheet density (calculated within a height of 2 nm) averaged over the entire substrate surface. The inset shows the SEY as a function of incident electron energy.

The discharge is initiated by strong cathode field emission. The typical average breakdown field in NPDs ranges from 0.1 to 1 V/nm and is further enhanced near the cathode triple point (also referred to as the cathode triple junction), namely the local junction where the cathode, substrate, and surrounding gas meet [77, 78, 79], leading to intense field emission. Field emission electrons are accelerated by the applied electric field. Their initial trajectories follow an angular distribution [80], and a fraction of them strike the substrate with high energy (>∼50 eV, shown in the left intersection in the inset of Figure 2d), producing secondary electron emission. The secondary emission yield, δ_e_, defined as the number of secondary electrons (SEs) created by one incident electron, must be higher than one to initiate the discharge [81, 82, 83]. When δ_e_ > 1, Positive surface charges are accumulated on the substrate, attracting subsequent electrons to the substrate and inducing further secondary electron emissions, known as the secondary electron emission avalanche [84, 85, 86].

The complete SEEA process in a nanogap is reproduced by a particle‐in‐cell simulation with a finite‐element method solver for the electrostatic Poisson equation, as shown in Figure 2 and Video S1. The simulation setup, detailed in Section 5.3, is consistent with the real device design, where the cathode (left) and anode (right) are connected by a substrate.

The results in Figure 2a,b show that the SEEA propagates from the cathode to the anode and covers the entire substrate surface once fully developed, accompanied by the expansion of an electron layer with an ultra‐high density in the order of ∼10^25^ m^−3^. The time required for SEEA development is 0.2 ps on a 500 nm substrate (shown in Figure 2a,b) and 0.1 ps on a 250 nm substrate (shown in Figure 2c,d and Video S1), suggesting nearly constant development speed. During SEEA development, the positive surface charge region on the substrate expands from the cathode to the anode, as shown in Figure 2c. Once SEEA is fully developed, the surface charge distribution becomes uniform beyond ∼50 nm from the cathode, while the surface charge density increases within the first 50 nm near the cathode.

After the SEEA is fully established, a quasi‐equilibrium is achieved, where the net SEY of the entire substrate equals one, and both the surface charge density and the electron sheet density remain constant, indicating a balance of charging, as shown in Figure 2c. A UES is formed above the substrate surface within a height of 2 nm, as shown in Figure 2c. Notably, the present model suggests that this equilibrium may persist for a much longer duration in high vacuum if the outgassing effect is weak, which remains to be experimentally verified. Under atmospheric pressure, however, the equilibrium persists only for a sub‐picosecond duration before the ionization avalanche begins.

The UES formation is further elaborated below. Although the electron sheet density is comparable to that of heavily n‐doped semiconductors, electrons in the UES are fundamentally different from charge carriers in solids. These electrons are confined near the substrate by the vertical electric field of the positive surface charges and travel toward the anode via successive secondary electron emission (see the multimedia file Video S1 for visualized electron trajectories). As such, the UES is not a conductive medium and carries only a low current despite its high density. Therefore, its formation does not in itself constitute the breakdown. This also explains why a comparison of carrier density with conventional solid‐state devices does not imply similar output power. In solid‐state devices, electrical power is limited by bulk carrier transport, parasitic and output capacitance, carrier saturation velocity, and critical electric‐field constraints. In contrast, in the NPD, the UES acts only as a copious source of seed electrons, enabling the ionization avalanche to develop even when the gap length is smaller than the Townsend limit $d_{\mathrm{gap},\min}^{\mathrm{Townsend}}$. The much higher output power arises only after the ionization avalanche triggers the ultrafast nano‐plasma switching and allows rapid discharge of the stored energy into the resonator and load. Additional numerical simulation of the post‐SEEA stage further confirms that the SEEA‐generated seed electrons rapidly initiate an ionization avalanche, producing nano‐plasmas near the substrate surface within picosecond timescale (Figure S2).

An analytical model of the UES‐driven ionization avalanche was developed and compared with the simulation results for validation. The substrate surface charge density (σ_s_), electron sheet density (*n*_e,SEEA_), and thickness (*h*_SEEA_) are expressed as follows (derivations are provided in Equation S2):

(2) $$\sigma_{s}=\frac{\left(1+\varepsilon_{r}\right)\varepsilon_{0}}{\sqrt{0.5\left(\frac{\varepsilon_{e,1}}{\varepsilon_{e,0}}-1\right)}}E_{x}$$

(3) $$n_{e,\mathrm{SEEA}}=\frac{4\left(1+\varepsilon_{r}\right)\varepsilon_{0}}{\varepsilon_{e,1}-\varepsilon_{e,0}}E_{x}^{2}$$

(4) $$h_{\mathrm{SEEA}}=\frac{\varepsilon_{e,0}}{2eE_{y}}=\frac{\varepsilon_{e,0}}{2eE_{x}}\sqrt{0.5\left(\frac{\varepsilon_{e,1}}{\varepsilon_{e,0}}-1\right)}$$

Here, ε_r_ and ε_0_ are substrate relative permittivity and vacuum permittivity, ε_e,1_ is the electron incident energy that generates one SE, ε_e,0_ is the initial energy of SE, and *E*_x_ is the applied electric field.

**FIGURE 3:**
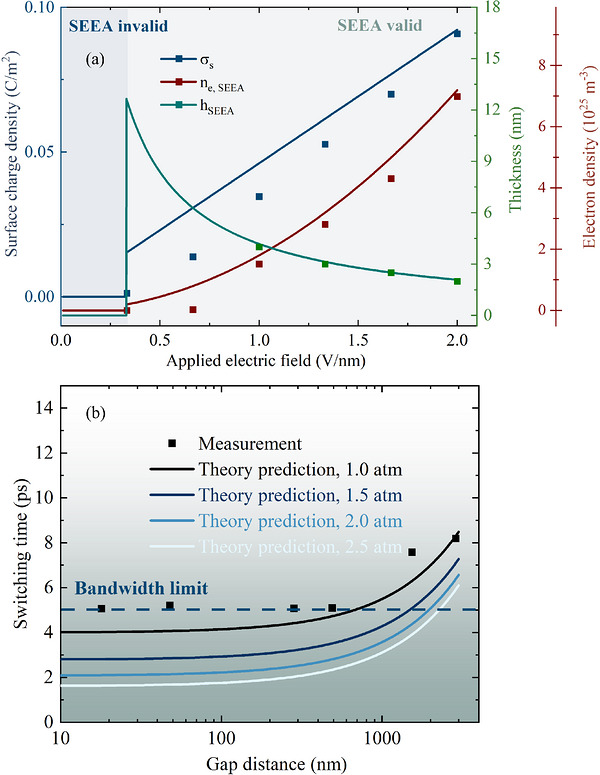
Framework validation with theory, simulation, and experiment. (a) Surface charge density, electron sheet density, and thickness predicted by theory of Equations (2)‐(4) (solid lines) and simulation (symbols) with varied electric field. The lower electric field range, where the SEEA model becomes invalid due to δ_**e**_<1 and negative surface charge accumulation on the substrate is indicated by the shaded area. (b) Switching time given by the theory of Equation (5) and experimental measurements with varying gap length in ambient air. Theory predictions with increasing air pressure and the measurement limit are indicated.

Equations (2)‐(4) suggest that the surface charge density, electron sheet density, and thickness scale with *E*_x_, $E_{x}^{2}$, and $E_{x}^{-1}$, with typical values of 0.01–0.1 C/m^2^, 10^25^ m^−3^, and 1–10 nm, respectively. The theoretical predictions are validated against simulation results obtained from a scan of the applied electric field, as shown in Figure 3a. The predictions and simulations are consistent at high *E*_x_ values, while discrepancies increase at lower *E*_x_. The theory is invalid when negative surface charges accumulate on the substrate for *E*_x_ lower than 0.3 V/nm, shown in the blue region in Figure 3a, as field emission electrons strike the substrate with an SEY lower than one and result in negative surface charge accumulation. This repels the subsequent electrons, preventing SEEA and THz emission.

After SEEA is fully established on the substrate, the UES initiates an ultrafast ionization avalanche on the picosecond scale, which is critical for the high THz power. The time interval between the full SEEA development and breakdown can be expressed by the following expression (derivations are provided in Equation S3):

(5) $$t_{\mathrm{on},2}=\frac{d}{v_{e,\mathrm{drift}}}+\frac{1}{\alpha_{T}\bar{v_{e}}-\tau_{\mathrm{diff}}^{-1}}\ln\left(\frac{n_{e,\mathrm{crit}}}{f_{\mathrm{ion}}n_{e,\mathrm{SEEA}}}\right)$$

Here *d* is the gap length, *v*_e,drift_ is the electron drift velocity, which depends on the electric field and the electron‐neutral collision cross section, α_T_ is the first Townsend coefficient of air, $\bar{v_{e}}$ is the mean electron velocity, τ_diff_ is the characteristic loss time for diffusion away from the substrate, *n*_e,crit_ is the critical plasma density at which breakdown is considered to occur, and *f*_ion_ = α_T_ *l*_x_ is the probability that a SE can induce ionization during its trajectory *l*_x_. The critical condition for ionization avalanche is $\alpha_{T}\bar{v_{e}}-\tau_{\mathrm{diff}}^{-1}>0$, meaning that the UES‐induced ionization rate must surpass the diffusion loss rate in order to accumulate net space charge. Equation (5) indicates that *t*_on,2_ increases linearly with gap length and decreases with higher UES density. Note that the total switching time is the sum of *t*_on,1_ (SEEA development) and *t*_on,2_ (ionization avalanche), with *t*_on,1_ ≪ *t*_on,2_. The calculated *n*_e,SEEA_, *l*_x_, and *f*_ion_ for a range of substrate materials, along with the adopted numerical parameters, are provided in Equation S4.

The total switching time as a function of gap length, as predicted by the theory, is compared with the measurements in Figure 3b. The theoretical predictions of atmospheric air agree with the experimental results within the measurement limit, and suggest that the actual NPD switching time could be faster than can be experimentally resolved. Note that our present setup allows up to 0.3‐0.5 THz time‐domain measurement.

In addition, theory predicts that the switching speed further increases with higher air pressure, due to higher α_T_. In atmospheric air, a gap length below ∼1 µm is required to ensure THz generation, whereas THz could be generated with larger gap lengths if the NPD is encapsulated in a pressurized cavity. More detailed analyses of the NPD experiments with varied gap lengths were provided in our previous works [29, 45].

### 2.2 Optimizing Substrate Material SEY for Enhanced THz Generation

The nanogap air breakdown framework proposed in Section 2.1 suggests that the discharge initiation critically depends on the secondary emission yield of the substrate material. The lower electric field bound of discharge initiation is given by ε_e,1_ ≤ *eE*_x,SEEA, min_ *d*, which requires the accumulation of positive surface charge on the substrate. The value of ε_e,1_ is typically determined by two characteristic SEY parameters, δ_e,max_ and ε_max_ [87, 88], representing the peak SEY and its corresponding incident electron energy, respectively, as shown in the inset of Figure 2d.

**FIGURE 4:**
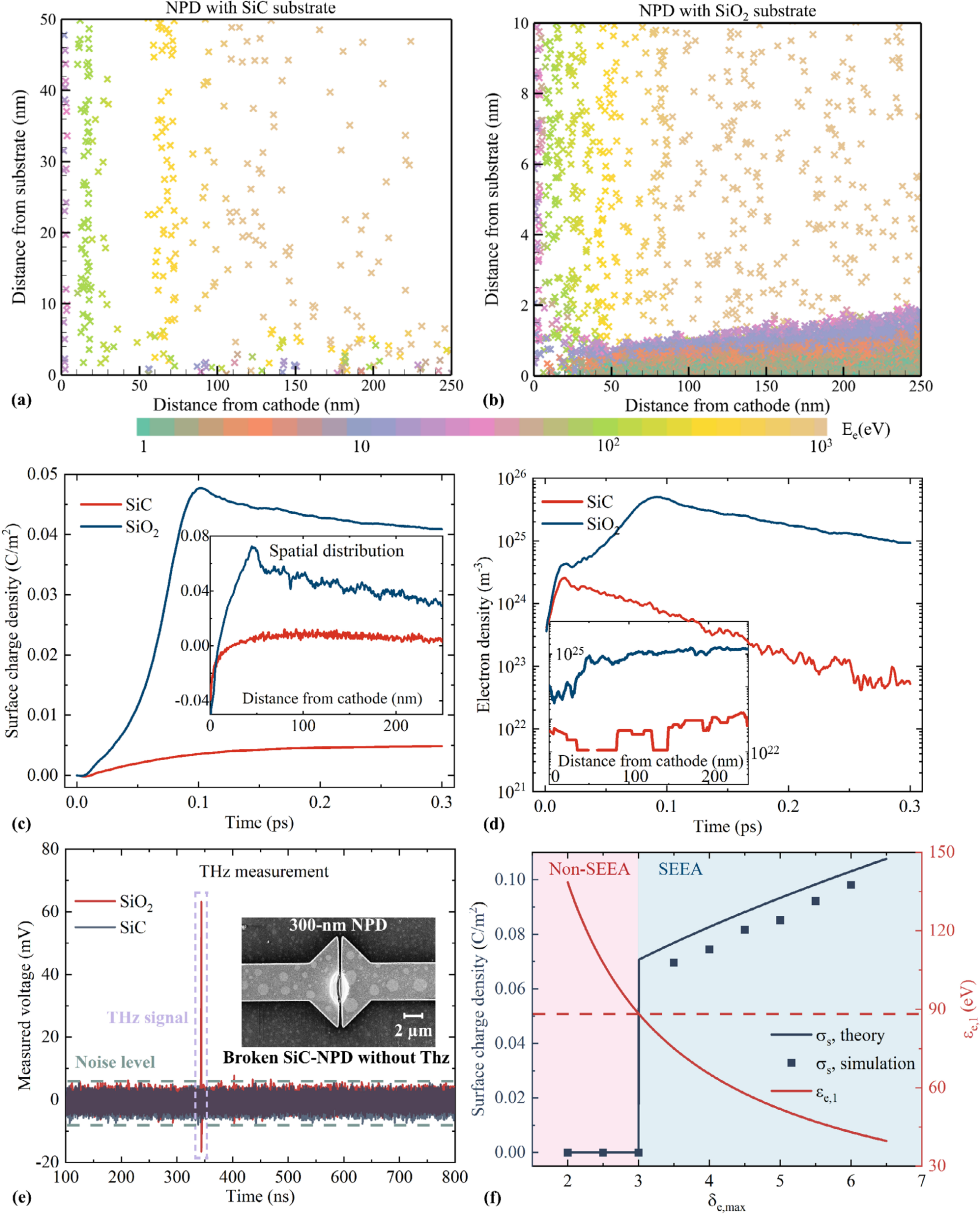
Simulation and experiment of NPDs with different substrate materials. (a) Converged electron distribution with a low‐SEY SiC substrate. No SEEA is formed. (b) Converged electron distribution with a high‐SEY SiO_2_ substrate. SEEA is formed. (c) Time evolution of the averaged surface charge density with SiC and SiO_2_ substrates. The inset shows the spatial distribution of surface charge density at convergence, where a low‐SEY substrate leads to negative surface charges. (d) Time evolution of the averaged electron sheet density with SiC and SiO_2_ substrates. The inset shows the converged spatial distribution of electron density. (e) Measured signals of NPDs with SiO_2_ and SiC substrates. The THz signal was only measured with SiO_2_ substrate and is highlighted. The inset shows the damaged NPD with a SiC substrate and a 300 nm gap length, where no THz signal was measured. (f) Simulated surface charge density and calculated ε_**e**,1_ with increasing δ_**e**,**max**_, using an ε_**max**_ of 500 eV. The threshold ε_**e**,1_ for SEEA initiation is indicated by shaded areas.

Substrates with low δ_e,max_ and high ε_max_ exhibit low SEY and high ε_e,1_, which requires higher *E*_x,SEEA, min_ for discharge initiation. To explore the effects of substrate SEY on the discharge initiation, simulations using substrates with high and low SEY (SEY data are shown in Table 1), namely SiO_2_ and SiC, were performed. Simulation results suggest that SEEA forms above the NPD with a SiO_2_ substrate but not with a SiC substrate, as shown in Figure 4a,b. The low‐SEY SiC substrate leads to an extended negatively charged region near the cathode in the SiC case, which repels most field emission electrons and prevents the discharge initiation, as shown in Figure 4c. Consequently, the NPD with a SiC substrate results in a much lower electron density at the substrate than with SiO_2_, preventing SEEA and THz generation, as shown in Figure 4d.

**TABLE 1:** Substrate SEY parameters and THz measurement results.

Material	δ_e,max_	ε_max_(eV)	ε_e,1_(eV)	THz emission
Sapphire	6.0	608	52	Yes
LiF	6.0	600	51	Yes
MgF_2_	6.1	680	57	Yes
SiO_2_	4.35	420	50	Yes
SiC	2.0	400	111	No
GaN	2.5	500	108	No
Diamond	2.9	890	163	No

For NPDs with low‐SEY substrates and high *E*_x,SEEA, min_ which exceeds the breakdown field of the bulk substrate, SEEA cannot occur, and a destructive breakdown of the substrate takes place, causing irreversible device damage and preventing THz emission. This is validated by experimental measurements of NPDs with SiO_2_ and SiC substrates shown in Figure 4e. THz was measured from NPDs with a SiO_2_ substrate but not with a SiC substrate, consistent with the simulation results. A broken NPD with low‐SEY SiC substrate material, which did not produce THz, is shown in the inset of Figure 4e. This irreversible failure is fundamentally different from the behavior of high‐SEY‐substrate NPDs, where repetitive switching and THz generation are possible.

The transition from a non‐SEEA regime with a slightly negatively charged substrate to an SEEA regime with a positively charged substrate is illustrated by the simulation with increasing δ_e,max_ at fixed ε_max_ in Figure 4f, where the threshold ε_e,1_ for SEEA initiation is predicted to be approximately 90 eV. The predicted SEY‐THz dependency is validated with more NPDs fabricated with different substrate materials, including sapphire, LiF, MgF_2_, GaN, and diamond, with the measurements of all seven types of NPD summarized in Table 1 and Equation S5.

It is found that NPDs fabricated on sapphire, LiF, MgF_2_, and SiO_2_ substrates with ε_e,1_ of approximately 50‐60 eV (high SEY) can produce THz emission, whereas those fabricated on SiC, GaN, and diamond substrates with ε_e,1_ higher than 100 eV (low SEY) cannot produce THz. Therefore, it is concluded that substrate materials with high SEY facilitate THz generation by enabling SEEA formation, whereas low‐SEY substrates suppress SEEA and consequently do not support the ultrafast switching required for measurable THz emission.

### 2.3 Optimum Range of NPD Gap Length for THz Generation

In Section 2.1, it has been shown that decreasing the gap length leads to faster switching and favors THz generation, whereas large gaps, above ∼ 1 µm, result in the output signal being too slow to produce THz. This determines the upper limit for the NPD gap length. In this section, we will demonstrate a lower limit of the NPD gap length for THz generation based on the discharge initiation condition, and define the optimal range of NPD gap length.

In the simulations of Figure 2a,b) and Figure 4b, it is observed that UES does not cover a region of approximately 10–20 nm near the cathode after it fully develops. The scale of this electron‐depleted region is denoted as *d*_void_. This region arises from the initial trajectories of the field emission electrons, as only a limited fraction of them with an initial direction toward the substrate are sufficiently accelerated by the applied electric field before their arrival at the substrate can contribute to the SEEA. Consequently, reducing the gap length below *d*_void_ will prevent discharge initiation and THz emission, consistent with the measurement in Figure 3b.

**FIGURE 5:**
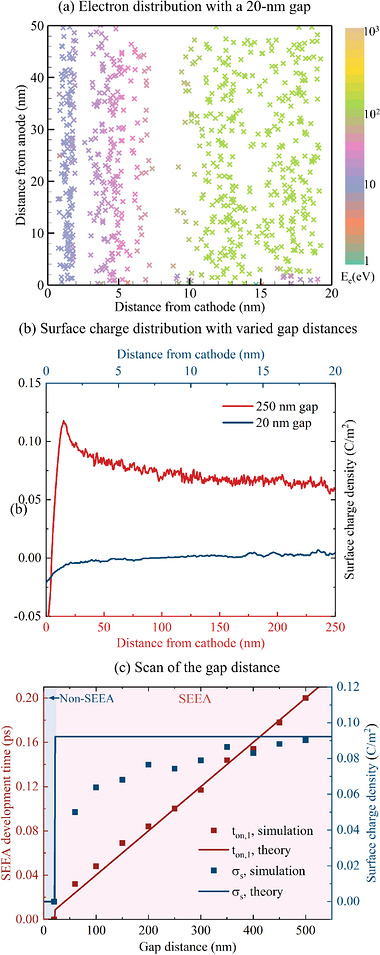
Simulation results of NPD with varied gap lengths. (a) Electron distribution with a gap length of 20 nm. No SEEA is formed. (b) Surface charge density distribution. The 20‐nm substrate accumulates negative surface charges. (c) Variation of surface charge density and the SEEA development time with increasing gap lengths. The threshold gap length for SEEA initiation is marked by the shaded areas.

The above analysis was validated by numerical simulations with varied gap lengths. First, simulations of a 20‐nm gap were performed and compared with the 250‐nm gap, shown in Figure 5a,b. The simulation results suggest that the discharge cannot initiate when the gap length decreases to 20 nm, as shown in Figure 5a. This is due to negative surface charges covering the whole substrate with a 20 nm gap length, as shown in Figure 5b. A simulation scan of the gap length is performed and is shown in Figure 5c. The simulated surface charge density is close to the theory prediction at gap lengths above 250 nm, and the discrepancy becomes larger as the gap length decreases, due to the fact that the weakly charged near‐cathode region contributes to a larger fraction of the averaged surface charge density. A sharp transition at a gap length below 20 nm is observed, where no SEEA is formed, consistent with the measurements.

It should be noted that the present NPD is intentionally designed to favor electron–substrate collision, so that SEEA and breakdown can still occur at very small gap lengths. This is different from nanochannel devices [89, 90], in which field emission electrons are transported ballistically, and collisions with the substrate are avoided even when the gap length exceeds *d*_void_.

In summary, the optimum NPD gap length is defined by two limits. The upper limit of ∼1 µm to ensure emission within the THz range, and the lower limit of ∼20 nm to ensure discharge initiation.

## 3 Discussions and Outlook

Section 2 established the first nanogap air breakdown framework for NPD‐based THz generation. In this section, the potential relevant applications in other established and emerging nanoscale applications are discussed.

First, SEEA and UES formation are unfavorable for nanogap devices relying on ballistic electron transport, where any breakdown must be prevented, such as the nanoscale air channel device [30, 91, 92, 93, 94]. Its typical gap length is shorter than that of NPDs, but not necessarily lower than *d*_void_. Therefore, to prevent the undesirable SEEA‐driven breakdown, the device design should avoid ballistic electron impact with the substrate surface, and a low‐SEY substrate should be selected if the ballistic electrons cannot be fully prevented.

Second, the present SEEA and UES framework suggests possible implications for high‐vacuum operation, where the atmospheric ionization avalanche observed in air would be suppressed. If experimentally confirmed, plasma‐free dense electron‐sheet behavior could inspire new vacuum nanoelectronic devices for ultrafast electron optics [95], contactless gating of low‐dimensional semiconductors [96], and high‐frequency systems [97]. SEEA under vacuum has been reported in micrometer‐scale devices such as ion‐trap chips [98, 99, 100], as well as in larger‐scale (millimeter and above) switches [101, 102] and insulators [103, 104, 105]. Further studies are required to determine whether a similar mechanism can occur in the present nanogap geometry under vacuum.

Additionally, the compact NPD design and its operation mechanisms make it inherently compatible with self‐powered systems [106, 107, 108]. NPDs operate in pulsed mode and require ∼100 V breakdown voltage, which matches well with the high‐voltage, low‐current output of triboelectric nanogenerator (TENG) by combining it with an energy‐storage capacitor. Recent studies have demonstrated that TENGs can successfully drive atmospheric micro‐plasmas with gap lengths below 1 mm in the form of dielectric barrier discharge, plasma jet, glow, spark, and corona discharges, for applications including micro‐plasma transistors, chemical activation, conversion, and decontamination [109, 110]. Extending the triboelectric micro‐plasma system to the nanoscale is feasible and favorable, as nano‐plasma requires lower breakdown voltage than micro‐plasma, according to Figure 1c. Building a triboelectric nano‐plasma system could enable self‐powered, on‐chip THz generation.

Lastly, device lifetime is an important engineering consideration for NPD and has been investigated in previous work. Available studies show that robustness can be improved by increasing electrode thickness [29, 111], which reduces degradation under repetitive operation, and more recent results further indicate that replacing tungsten with GaN electrodes can provide additional lifetime enhancement [48]. These observations suggest that device lifetime can be improved through optimization of electrode geometry and material. The demonstrated repeatability under prolonged operation also suggests that the breakdown is unlikely to be caused by debris bridging or powder residue, which would be expected to produce unstable behavior or rapid failure.

## 4 Conclusion

In this work, we discovered that an ultra‐dense (10^25^ m^−3^) electron sheet confined within ∼10 nm of the substrate can be formed in a nanogap via the secondary electron emission avalanche mechanism, which drives picosecond nano‐plasma switching in air essential for the THz generation based on nano‐plasma devices. We established and validated a first‐of‐its‐kind nanogap air breakdown framework through dedicated theories, simulations, and experiments, achieving consistent agreement on the switching time, surface charge density, electron sheet density, and thickness. It is found that the SEEA requires a minimum electric field of ∼10^8^ V/m to initiate, and can fully establish on a ∼100 nm gap within ∼0.1 ps, leaving the substrate positively charged. SEEA provides sufficient electron sources to initiate a nanogap breakdown in ∼1 ps, where the switching speed increases with higher gap length and decreases with higher neutral pressure. Model‐based device optimization demonstrated that selecting substrate materials with high secondary emission yield can enhance the THz emission, and that a minimum gap length is required for SEEA initiation. These findings define the optimum nanogap length range under atmospheric pressure that ensures both discharge initiation and output within the THz range. The framework may have implications for a broader class of applications, including possible device concepts in high‐field vacuum nanoelectronics and the integration of NPD with TENG to build triboelectric nano‐plasma systems. Together, these findings validated NPDs as a feasible and scalable platform for on‐chip, high‐power THz sources compatible with established and emerging nanosystems.

## 5 Methods

### 5.1 Nano‐Plasma Device Fabrication Process

**FIGURE 6:**
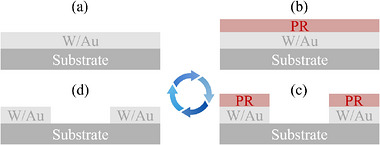
Fabrication process of the nano‐plasma device. (a) Metal deposition on the substrate by sputtering. (b) Resist coating. (c) Electron‐beam lithography. (d) Resist removal. Larger THz structures are patterned in a separate lithography step.

The fabrication process of the nano‐plasma device is illustrated in Figure 6. The devices were fabricated on two‐inch substrates with materials listed in Table 1. Standard planar nanofabrication techniques were employed. After substrate cleaning, a 3 nm chromium adhesion layer was first deposited, followed by a 250 nm gold layer, a 3 nm chromium adhesion layer, and a 150 nm tungsten layer. All metal depositions were performed by sputtering. The nanoscale discharge gap was defined by electron‐beam lithography using ZEP positive resist, followed by ion‐beam etching and resist removal. The larger THz structures were patterned separately using optical photolithography and etching. The resulting device integrates the nanogap with two coupled split‐ring THz resonators connected to a coplanar waveguide. The THz resonators were designed with an eigenfrequency of 0.4 THz, corresponding to the upper limit of our available measurement setup.

### 5.2 THz Measurement via Down‐Conversion and Power Estimation

For chip‐scale THz power measurements, the nano‐plasma device was operated under pulsed microwave excitation. A 100 ns microwave pulse generated by standard RF circuitry was applied to the device through a microwave ground–signal–ground probe. The nano‐plasma device was monolithically coupled to a 0.4 THz resonator and a 50 Ω output load. To improve output coupling and maximize the measurable THz power, a coupled‐resonator structure was used. In this design, the second resonator suppresses re‐absorption of the generated THz signal at the input by destructively interfering with the back‐propagating wave and isolating the resonant structure from the feed line, thus minimizing the reflected power to match with the 50‐Ω load. Different substrate dielectric constants were taken into account by tuning the resonator design. The resonator was designed at 0.4 THz because this frequency corresponded to the upper limit of our available THz measurement setup. The emitted THz wave packet was routed through an on‐chip waveguide attenuator (24 dB), a THz ground–signal–ground probe (≈4.5 dB), a frequency‐extender input waveguide (≈2.5 dB), the extender conversion loss (≈6 dB), and the intermediate‐frequency (IF) cable (≈3 dB) to a real‐time oscilloscope operating at ≈40 GHz. The measured IF amplitude, corrected for the known losses and IF bandwidth using a simulation [44] of a lossless mixer path, was mapped back to the THz port voltage to estimate the peak power at 0.4 THz.

All time‐domain traces were de‐embedded for the probe/cable chain using measured S‐parameters and then inverse‐filtered in the frequency domain to recover the device‐plane waveform. The same procedure was applied to both radiated/received paths and guided THz paths. For peak‐power estimates, the recovered device‐plane voltage across 50 Ω was used (*P*_peak_ = $V_{\mathrm{peak}}^{2}$/50Ω). For the 0.4 THz device, simulations established the IF‐to‐THz scaling for short wave packets, where an IF measurement of ∼2 V corresponds to ∼10 V at 0.4 THz and a peak power of ∼2 W at the 50 Ω THz port. A more detailed description of the THz measurement was provided in [44].

### 5.3 Particle‐in‐Cell Simulation Setup

The particle‐in‐cell numerical simulation employed a finite‐element method field solver and a 2D3V (two spatial dimensions, three velocity components) simulation space. The model self‐consistently accounted for field emission, secondary electron emission, electron motion, substrate surface charging, and the electrostatic field obtained from solving Poisson's equation. It was adopted from a millimeter‐scale vacuum discharge simulation code developed in our previous work [112], where dedicated benchmarks were performed to validate the code.

**FIGURE 7:**
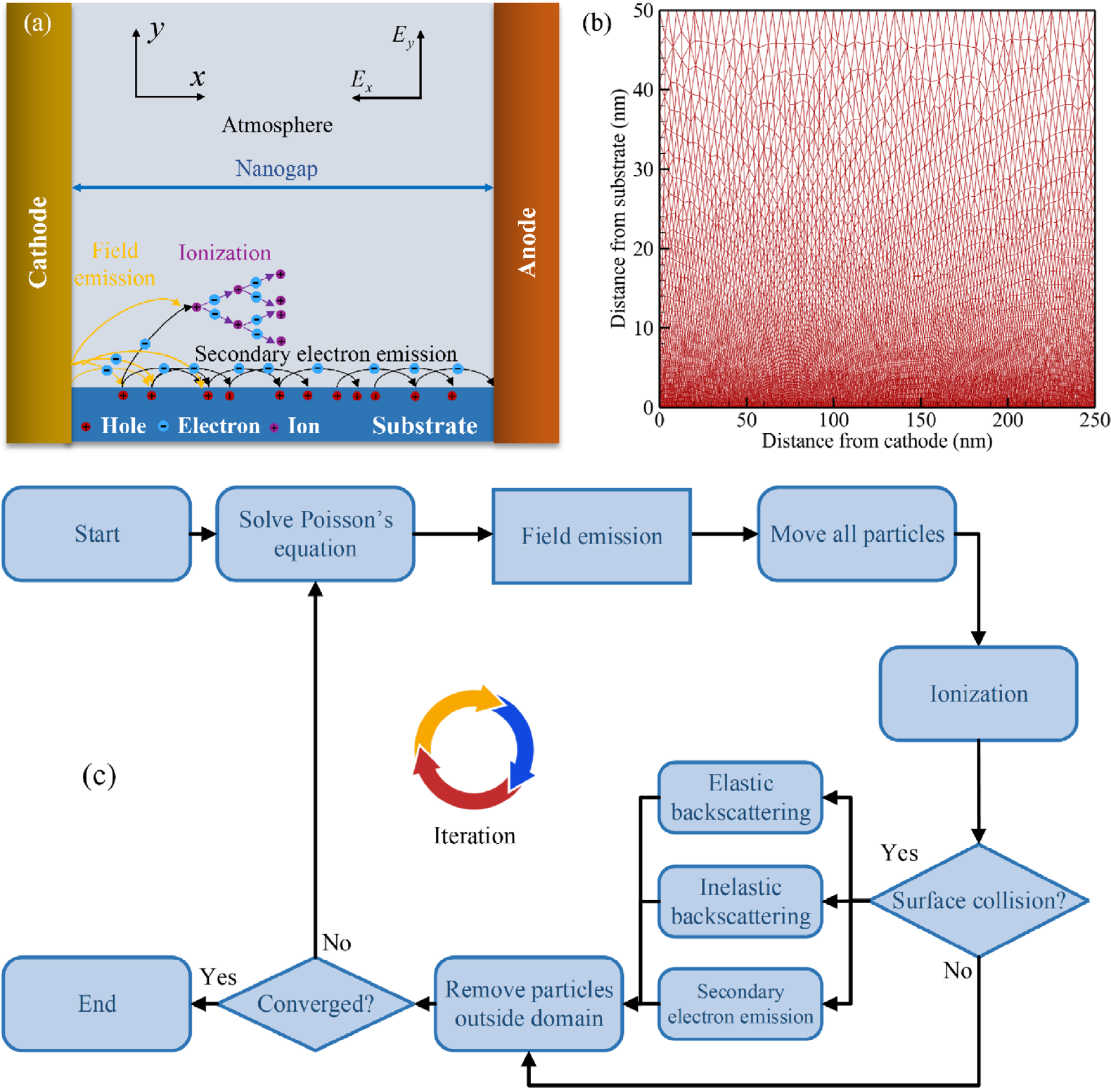
Simulation setup. (a) Schematic of the simulation domain. (b) Example of the simulation mesh based on the finite‐element method. (c) Flow chart of the program execution procedure.

The simulation domain comprises two planar metal electrodes separated by a sub‐µm discharge gap and a substrate bridging the gap, as shown in Figure 7a. Dirichlet boundary conditions were applied to the electrodes, with the cathode grounded and a voltage applied to the anode. Neumann conditions were applied to the upper and lower domain boundaries: the far‐substrate side was set to zero electric field, while the substrate side was assigned an electric field of $E_{y}=\frac{\sigma_{s}}{\left(1+\varepsilon_{r}\right)\varepsilon_{0}}$, determined by the substrate permittivity, ε_0_ε_r_ and the surface charge density, σ_s_.

Field emission electrons were injected from the entire cathode surface, with the emission current (*J*_FE_,) prescribed by the Fowler–Nordheim field‐emission formula [113, 114]:

(6) $$J_{\mathrm{FE}}=\frac{e^{3}}{8\pi h_{p}}\frac{E_{C}^{2}}{\phi w}\exp\left(-\frac{8\pi \sqrt{2m_{e}}}{3eh_{p}}\frac{\phi w^{3/2}}{E_{C}}\right)$$

Here *β* is the field‐enhancement factor, *h*_p_ is the Planck constant, *E*_c_(*y*) is the cathode field at vertical position *y*, ϕw is the work function of the cathode material. At each timestep, the number and location of field emission electrons were determined from the local current density evaluated on the cathode mesh. Emitted electrons had a fixed injection energy of 0.5 eV and followed a cosine angular distribution [115]. Three types of electron–substrate collisions were considered: secondary electron emission, elastic backscattering, and inelastic backscattering. The SEY (δ_e, SEE_) was calculated from empirical formulae as described in Equation S4. The elastic and inelastic backscattering yields were calculated from the following equations [116]:

(7) $$\delta_{e,\mathrm{elastic}}=r_{e}\delta_{e,\mathrm{SEE}}+\left\{\begin{array}{c} \delta_{e,\max}w_{1}e^{1-w_{1}}\\ \delta_{e,\max}\left(1+w_{2}\right)e^{-w_{2}} \end{array}\right.\begin{array}{c} \varepsilon_{e,\min}<\varepsilon_{e}<\varepsilon_{e,\max}\\ \varepsilon_{e}>\varepsilon_{e,\max} \end{array}$$

(8) $$w_{1}=\frac{\varepsilon_{e}-\varepsilon_{e,\min}}{\varepsilon_{e,\max}-\varepsilon_{e,\min}}$$

(9) $$w_{2}=\frac{\varepsilon_{e}-\varepsilon_{e,\max}}{\Delta \varepsilon }$$

(10) $$\delta_{e,\mathrm{inelastic}}=r_{i}\delta_{e,\mathrm{SEE}}$$

Here *r_e_* and *r_i_* are elastic and inelastic backscattering coefficients, ε_e_ is incident electron energy, ε_e,0_, ε_e, max_ and Δε are three constant coefficients controlling the elastic backscattering. Elastic collisions reversed the electron's vertical velocity while conserving its energy, whereas inelastic collisions re‐emitted the electron with energy uniformly sampled up to the incident value and with an isotropic direction. The change in surface charge density after each collision was given by:

(11) $$\Delta \sigma_{s}=\frac{w_{e}e}{\Delta x}\left(\delta_{e,\mathrm{SEE}}-1\right)$$

Here *w*_e_ is the particle weight (number of real electrons represented by a simulated electron), and Δ*x* is the mesh resolution in the *x* direction.

Electron trajectories were advanced with a leapfrog integrator under the influence of the space electric field. Space charges were distributed to the mesh grid via area‐weighted interpolation on triangular elements. The 2D Poisson equation was then solved at each timestep to calculate the electric field. To enhance simulation precision, the mesh density was increased near the substrate and electrode surfaces, where high space‐charge density and strong field gradients appeared, as shown in Figure 7b. The sequence of simulation steps is summarized in the flow chart in Figure 7c.

The code was implemented in Python with the Numba JIT compiler, and particle motion was parallelized across several CPU cores via the Message Passing Interface (MPI). FEM meshes were generated using the Gmsh module [117]. The timestep and mesh resolution followed the Courant–Friedrichs–Lewy condition and were limited by the Debye length and electron frequency. The particle weight was chosen to ensure that more than 10^6^ particles were simulated in the SEEA scheme.

Key simulation parameters are listed in Table 2.

**TABLE 2:** Detailed list of simulation parameters.

Parameter	Value	Definition
*l*	20–500 nm	Substrate length/gap length
*h*	50 nm	Height of simulation domain
ϕw	4.5 eV	Electrode material work function
ε_e,0_	2.89 eV	Initial energy of SE
ε_e, min_	0.01 eV	Minimum electron energy for elastic backscattering
ε_e, max_	7.5 eV	Electron energy for maximum backscattering probability
Δε	3 eV	Attenuation coefficient for elastic backscattering
*r*_e_	0.03	elastic backscattering coefficient
*r*_i_	0.07	inelastic backscattering coefficient
Δ*x*_bot_	∼0.01 nm	Grid size at the bottom boundary of the FEM mesh
Δ*x*_top_	∼1 nm	Grid size at the top boundary of the FEM mesh
Δ*t*	10^−6^ ps	Simulation time step
*w*_e_	10^5^–10^6^	Macro particle weight

## Author Contributions

**Guangyu Sun**: conceptualization, investigation, writing – original draft, writing – review and editing, visualization, validation, methodology, software, formal analysis, data curation, resources. **Mohammad Rezaei**: conceptualization, investigation, writing – original draft, methodology, validation, visualization, writing – review and editing, formal analysis, data curation, resources. **Yuheng Hu**: software, visualization, data curation. **Guan‐Jun Zhang**: supervision, writing – review and editing. **Elison Matioli**: conceptualization, investigation, funding acquisition, methodology, validation, visualization, writing – review and editing, software, formal analysis, project administration, data curation, supervision, resources.

## Conflicts of Interest

The authors declare no conflict of interest.

## Supporting information




**Supporting File 1**: advs75300‐sup‐0001‐SuppMat.docx.

**Supporting File 2**: advs75300‐sup‐0002‐MovieS1.mp4.

## Data Availability

The data that support the findings of this study are available from the corresponding author upon reasonable request.
