# Supplementary material for: High‐Power Terahertz Emission from Picosecond Nano‐Plasma Switching Driven by Secondary Electron Emission Avalanche
Source: Adv Sci (Weinh). 2026 Apr 21;13(38):e75300. doi: 10.1002/advs.75300 (PMC13335563; doi:10.1002/advs.75300)
Supplement: Supplementary file 1 — Supporting File 1: advs75300‐sup‐0001‐SuppMat.docx. [file ADVS-13-e75300-s002.docx]

Supplementary Information

High-power terahertz emission from picosecond nano-plasma switching driven by secondary electron emission avalanche

Guangyu Sun ^1^, Mohammad Rezaei ^1^ Yuheng Hu^2^, Guanjun Zhang^2^, and Elison Matioli ^1^ *

^1^ Power and Wide-band-gap Electronics Research Laboratory (POWERlab), Institute of Electrical and Micro-Engineering, École Polytechnique Fédérale de Lausanne (EPFL), Lausanne, Switzerland

^2^ School of Electrical Engineering, Xi’an Jiaotong University, Xi’an, Shaanxi 710049, China

KEYWORDS: nano-plasma; terahertz; nanoelectronics; secondary electron emission; field emission.


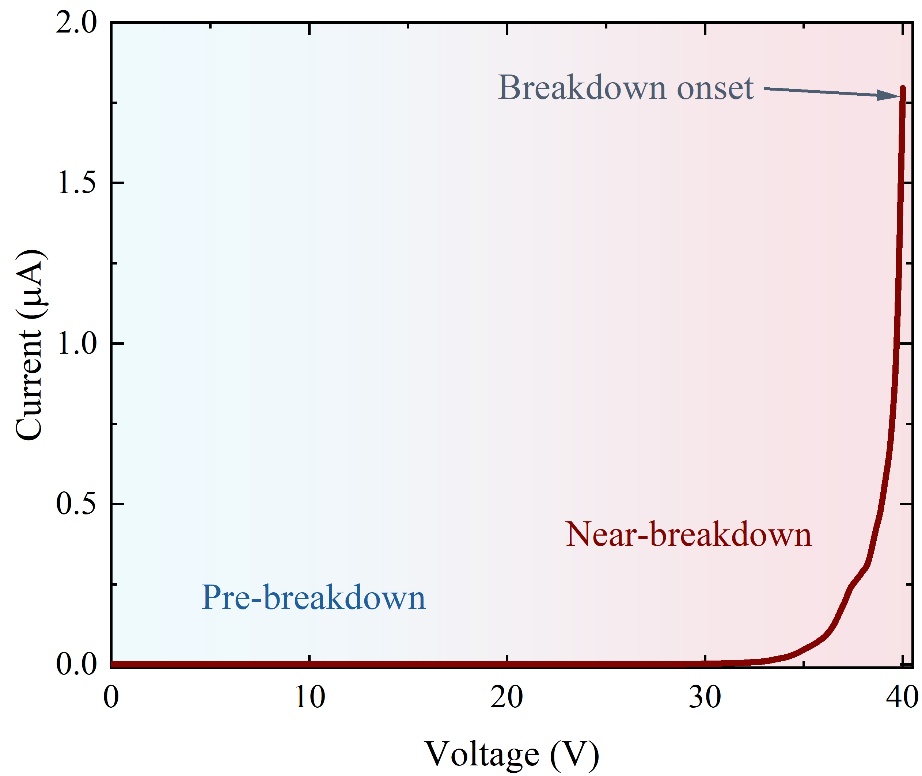


Figure S-1. Representative pre-breakdown I–V characteristic of the SiO_2_-based nano-plasma device measured by a slow DC voltage sweep on a probe station. The device remains in a low-current pre-breakdown state over most of the voltage range, followed by a near-breakdown current increase and finally the onset of breakdown when the applied voltage surpasses a threshold breakdown voltage.


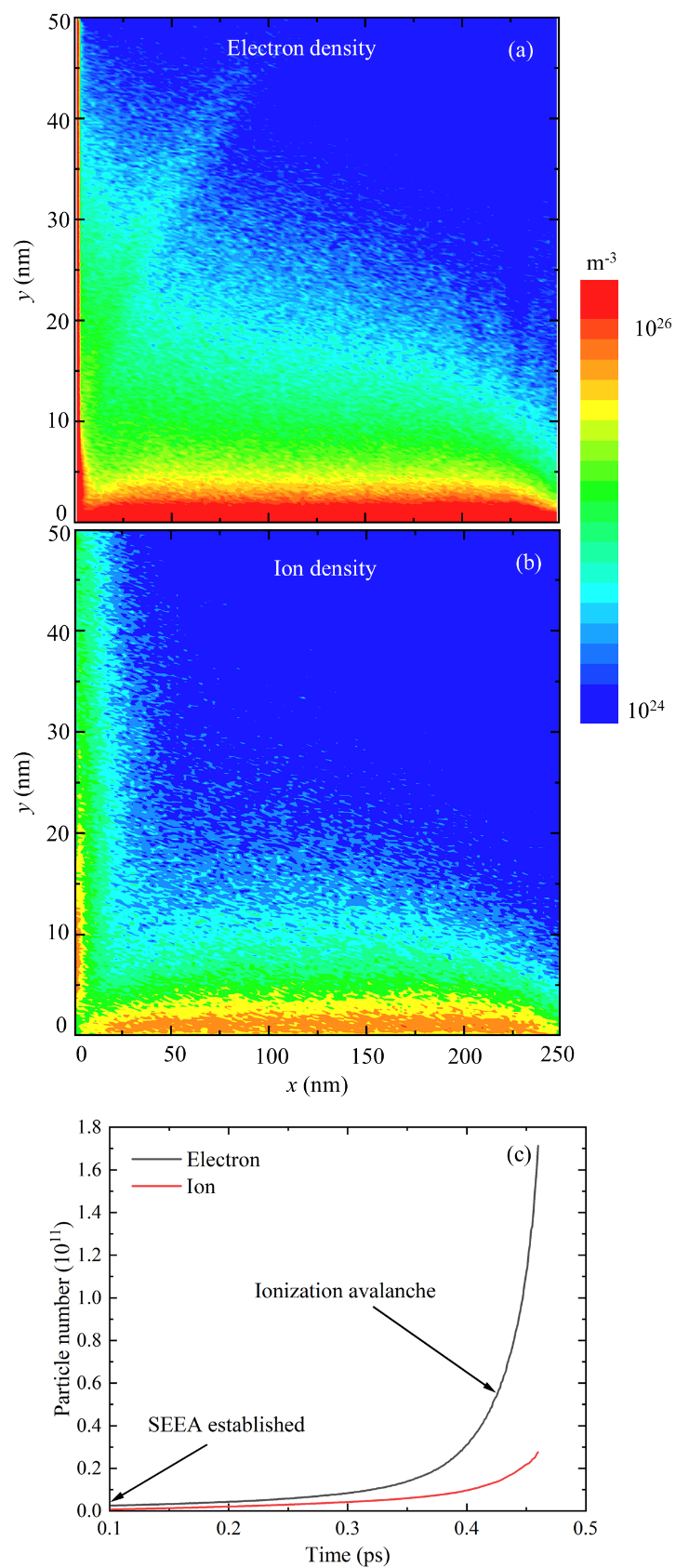


Figure S-2. Numerical simulation of the initial stage of nano-plasma formation after SEEA establishment. (a) Spatial distribution of electron density during the post-SEEA ionization-avalanche stage. (b) Spatial distribution of ion density during the same stage. (c) Time evolution of the total electron and ion populations in the simulated region during the first 0.5 ps after SEEA establishment. The rapid growth of both populations indicates the onset of ionization avalanche and ultrafast nano-plasma formation near the substrate surface.

1. **Spectral analyses of the terahertz generation by NPD**

The picosecond switching of an NPD can be modeled by a Hertzian dipole with a charge separation distance of $d_{\mathrm{gap}}$ which is the NPD gap distance. The Hertzian dipole model is valid if $kd_{\mathrm{gap}}\ll1$ with the wave vector $k=\frac{2\pi}{\lambda}=\frac{2\pi f}{c}$, here $\lambda$ is the wavelength, $f$ is the radiation frequency and $c$ is the speed of light. The condition is fulfilled for NPD for an eigenfrequency $f_{0}$ of 0.4 THz and $k$ of 10^4^ m^-1^, the condition $kd_{\mathrm{gap}}\ll1$ is satisfied for an NPD with $d_{\mathrm{gap}}$ ~ 100 nm.

For a current $I\left( t \right)$ flowing across an effective gap length $d_{\mathrm{gap}}$, the dipole moment is:

$p(t) = d_{\mathrm{gap}} q(t)$

(S-1)

where $I\left( t \right)=\dot{q}(t)$. Since the radiated electric field (transverse component) is proportional to the time derivative of the current, the radiated field is proportional to the current ramp rate:

$E_{\mathrm{rad}}\left( t \right)\propto\ddot{p_{\perp}}=d_{\mathrm{gap}}\dot{I}\left( t \right)$

(S-2)

And therefore, the following scaling laws of the total radiated power and THz power are obtained:

$P_{\mathrm{rad}}\left( t \right)\propto\left[ d_{\mathrm{gap}}\dot{I}\left( t \right) \right]^{2}$

(S-3)

$P_{THz}(f_{0})\propto{(d_{\mathrm{gap}}2\pi f_{0})}^{2}\left| \tilde{I}(2\pi f_{0}) \right|^{2}$

(S-4)

Here, $f_{0}={(LC)}^{-0.5}$ is the LC resonator eigenfrequency.

Assuming an exponential current turn-on edge:

$I\left( t \right)=\Delta I(1-e^{t/\tau})$

(S-5)

Here $\tau$ is the switching time constant. The 10–90% switching time satisfies $t_{on,10-90\%}\approx2.2\tau$. Performing Fourier transform, it is obtained that:

$\left| \tilde{E_{\mathrm{rad}}}(2\pi f_{0}) \right|\propto\frac{d_{\mathrm{gap}}\Delta I}{\sqrt{1+{(2\pi f_{0}\tau)}^{2}}}$

(S-6)

Impose an allowable amplitude loss α, the minimum switching time for generating radiation at $f_{0}$ is derived:

$t_{on,10-90\%}\leq\frac{1.1}{\pi f_{0}}\sqrt{\alpha^{-2}-1}$

(S-7)

For $f_{0}$=0.5THz, $t_{on,10-90\%,max}$ is equal to 0.7ps, 1.2ps, 2.1ps, for $\alpha$ being equal to -3, -6, -10 dB, respectively.

1. **Derivation of the linear relation between parallel and vertical electric field**

We consider a quasi-equilibrium after SEEA is fully established and covers the substrate surface, where a secondary electron (SE) generated from the substrate is attracted by the positive surface charge, $\sigma_{\mathrm{SEEA}}$, and restrikes on the substrate with an energy $\varepsilon_{e,1}$, which corresponds to the energy required to produce exactly one SE. The parabolic trajectory of SEs is shown in Figure S-3.

Figure S-3. The SE’s parabolic trajectory with emission angle and axis definitions.

The initial SE direction follows cosine distribution, with the projection of SE flux on a solid angle $d\Omega=\sin\left( \alpha\right)\cdot d\alpha d\varphi$ proportional to $cos(\alpha)$:

$d\Gamma_{\mathrm{se}}=\Gamma_{\mathrm{se}}\frac{cos(\alpha)}{\pi}\cdot d\Omega$

(S-8)

Here $d\Gamma_{\mathrm{se}}$ the differential flux of SE. Equation (S-8) is equivalently expressed by the polar angle ($\alpha$) and azimuthal angle ($\varphi$) using the following probability distribution functions [1].

$f\left( \alpha\right)=\sin\left( 2\alpha\right),\alpha\in(0,0.5\pi)$

(S-9)

$g\left( \varphi\right)=\frac{1}{2\pi},\varphi\in(0,2\pi)$

(S-10)

The initial energy of SE is simplified as a constant value $\varepsilon_{e,0}$, whose value can be obtained by averaging over the empirical SE energy spectrum [2]. The SE lifetime is:

$\tau_{\mathrm{SE}}=2\frac{v_{y,0}}{a_{y}}$

(S-11)

where $v_{y,0}=\sqrt{2\varepsilon_{e,0}/m_{e}}cos(\alpha)$ is the initial SE velocity in *y* (vertical) direction, and $a_{y}=eE_{y}/m_{e}$ is the vertical acceleration which depends on the vertical electric field, $m_{e}$ is the electron mass. Averaging $v_{y,0}$ over the distribution functions in Equation (S-9), (S-10) yields $v_{y,0}=\frac{2}{3}\sqrt{2\varepsilon_{e,0}/m_{e}}$.

The electron leap distance in *x* direction during its entire lifetime is expressed as:

$l_{x}=v_{x,0}\tau_{\mathrm{SE}}+0.5a_{x}\tau_{\mathrm{SE}}^{2}$

(S-12)

Here $v_{x,0}=\sqrt{2\varepsilon_{e,0}/m_{e}}sin(\alpha)cos(\varphi)$ is the initial SE velocity in *x* (parallel) direction, and $a_{x}=eE_{x}/m_{e}$ is the parallel acceleration which depends on the parallel electric field.

Combining Equations (S-11), (S-12), the following relation between $E_{x}$ and $E_{y}$ is derived:

$0.25\left( \frac{\varepsilon_{e,1}}{\varepsilon_{e,0}}-1 \right)=\frac{E_{x}}{E_{y}}\sin\left( \alpha\right)\cos\left( \alpha\right)\cos\left( \varphi\right)+{(\frac{E_{x}}{E_{y}})}^{2}\cos^{2} \left( \alpha\right)$

(S-13)

Equation (S-13) is averaged over the distribution functions in Equation (S-9), (S-10), leading to a linear relation between $E_{x}$ and $E_{y}$ which only depends on the substrate secondary emission yield curve:

$\frac{E_{y}}{E_{x}}={[0.5(\frac{\varepsilon_{e,1}}{\varepsilon_{e,0}}-1)]}^{-0.5}=tan(\theta_{E})$

(S-14)

Here $\theta_{E}$ is the angle between vertical and parallel electric field.

1. **Theory of SEEA-induced breakdown**

The vertical electric field is mainly contributed by the substrate surface charges, with the following relation based on Gauss's law.

$E_{y}=\frac{\sigma}{(1+\varepsilon_{r})\varepsilon_{0}}$

(S-15)

where $\sigma$ is the substrate surface charge density, $\varepsilon_{r}$ is the substrate relative permittivity and $\varepsilon_{0}$ is the vacuum permittivity. Due to charge conservation, the total amount of charges of the substrate surface should be equal to that of the SEEA electron layer, with opposite charge polarity. The SEEA electron density is therefore expressed as:

$n_{e,SEEA}=\frac{\sigma}{eh_{\mathrm{SEEA}}}$

(S-16)

Here $h_{\mathrm{SEEA}}$ is the height of the SEEA electron layer, which is expressed as:

$h_{\mathrm{SEEA}}=\frac{\varepsilon_{e,0}\cos^{2} \left( \alpha\right)}{eE_{y}}$

(S-17)

Averaging over the distribution functions in Equation (S-9), (S-10) yields:

$h_{\mathrm{SEEA}}=\frac{\varepsilon_{e,0}}{2eE_{y}}=\frac{\varepsilon_{e,0}}{2eE_{x}tan(\theta_{E})}$

(S-18)

Combining Equations (S-15)-(S-17), and by averaging with the distribution functions in Equation (S-9), (S-10), the SEEA electron density is derived as:

$n_{e,SEEA}=\frac{2}{\varepsilon_{e,0}}(1+\varepsilon_{r})\varepsilon_{0}E_{y}^{2}\propto E_{y}^{2}\propto E_{x}^{2}$

(S-19)

Due to the high electron concentration (~10^25^ m^-3^), ionization becomes remarkable even in the nano-plasma device with a gap distance comparable or lower than the electron ionization mean free path under atmosphere pressure. However, it must be noted that SEEA electrons are not “free” electrons that cannot fully participate in the ionization avalanche. Their lifetime is limited by $\tau_{\mathrm{SE}}$, during which they travel a distance of $l_{x}$ in the *x* direction. Due to the short $l_{x}$, most SEEA electrons cannot induce ionization. The fraction of SEEA electrons participating in the ionization avalanche is:

$f_{\mathrm{ion}}=\alpha_{T}l_{x}$

(S-20)

Here $\alpha_{T}$ is the Townsend ionization coefficient, and $l_{x}$ averaged over the SE angular distribution is:

$l_{x}=\frac{\varepsilon_{1,0}-\varepsilon_{e,0}}{eE_{x}}$

(S-21)

During the SEEA-to-breakdown transition, electrons participate in the ionization avalanche while drifting from cathode to anode.

The ionization avalanche initiated by SEEA electrons is modelled by an exponential growth over time, including loss due to diffusion towards free boundary:

$n_{e,plasma}(t)=f_{\mathrm{ion}}n_{e,SEEA}e^{(\alpha_{T}\bar{v_{e}}-\tau_{\mathrm{diff}})t}$

(S-22)

Here $n_{e,plasma}$ is the plasma density, $\bar{v_{e}}$ is the average SEEA electron velocity, and $\tau_{\mathrm{diff}}$ is the characteristic diffusion loss time, which is expressed as:

$\tau_{\mathrm{diff}}=\frac{L^{2}}{\pi^{2}D_{e}}$

(S-23)

where $L$ is the nano-plasma device size, $D_{e}$ is the electron diffusivity. From Equation (S-22), the breakdown is possible only if $\alpha_{T}\bar{v_{e}}>\tau_{\mathrm{diff}}^{-1}$, from which a threshold breakdown electric field can be solved numerically. The breakdown voltage then scales linearly with the gap distance, due to a constant breakdown field.

The drift of electrons is simply modelled by a drift velocity proportional to $E_{x}$:

$v_{e,drift}=\mu_{e}E_{x}$

(S-24)

Here the electron mobility $\mu_{e}=en_{g}\sigma_{mom,e-n}v_{e}/m_{e}$, with $n_{g}$ the gas molecule density, $\sigma_{mom,e-n}$ the momentum transfer cross section, and $v_{e}$ the electron velocity.

The criterion for THz generation is when a critical electron density, $n_{e,crit}$, appears in anode, and sufficiently conductive current switches on the nano-plasma device. The time after the SEEA is fully established until the breakdown is calculated to be:

$t_{on,2}=\frac{d}{v_{e,drift}}+\frac{1}{\alpha_{T}\bar{v_{e}}-\tau_{\mathrm{diff}}^{-1}}ln(\frac{n_{e,crit}}{f_{\mathrm{ion}}n_{e,SEEA}})$

(S-25)

Here $d$ is the device gap distance. Note that the time of SEEA establishment is noted as $t_{on,1}$ (predicted by simulation), and the total nano-plasma switching time is $t_{\mathrm{on}}=t_{on,1}+t_{on,2}$.

1. **Numerical predictions of the picosecond nano-plasma switching theory**

The numerical values are implemented to equations derived in section 1 and 2, to validate the theories against simulation and measurement.

The secondary electron emission yield curve of a substrate material is characterized by the peak value ($\delta_{e,max}$) and the incident electron energy corresponding to this value ($\varepsilon_{e,max}$). Empirical SEY curves as a function of incident electron energy ($\varepsilon_{e}$) and angle ($\theta_{\mathrm{SE}}$) can be derived from these two parameters using dedicated empirical law [3]:

$\delta_{e,SEE}\left( \varepsilon_{e},\theta_{\mathrm{SE}} \right)=1.526\delta_{e,max}\left( 1+\frac{\theta_{\mathrm{SE}}^{2}}{2\pi} \right)\left[ 1-\exp\left( -z^{1.725} \right) \right]/z^{0.725}$

(S-26)

$z=1.284\varepsilon_{e}/[\varepsilon_{e,max}(1+\frac{\theta_{\mathrm{SE}}^{2}}{\pi})]$

(S-27)

By setting $\delta_{e}=1$ in the left branch of the SEY curve, the key parameter for SEEA theory $\varepsilon_{e,1}$ can be derived. The SEY curves and key SEY parameters of the substrate materials tested in this work are shown in Figure S-4 and Table S-1, respectively.


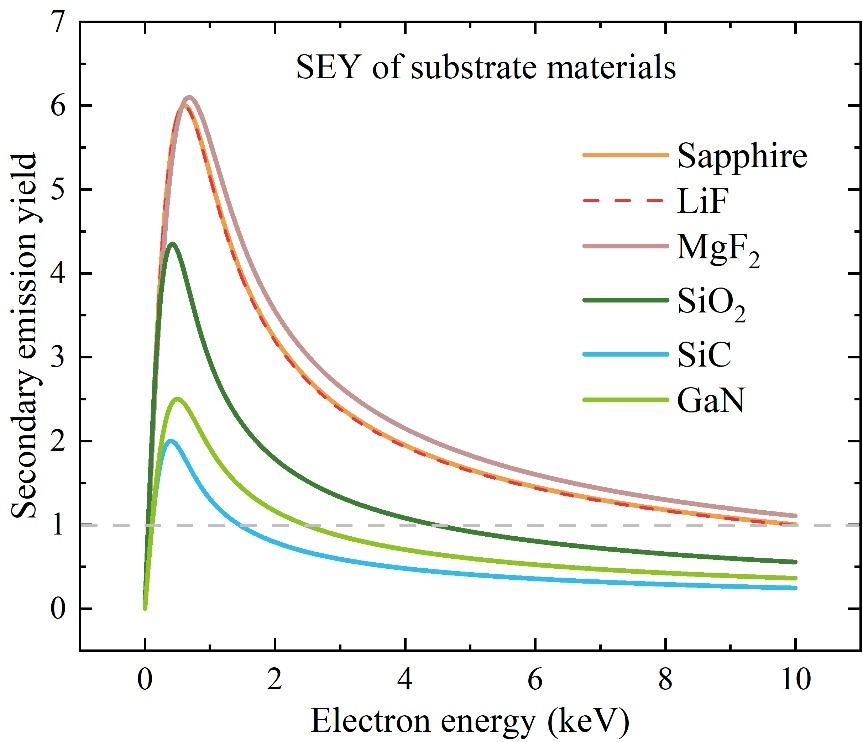


Figure S-4. Calculated secondary electron emission curve for tested substrate materials.

Table S-1. Characteristic parameters for secondary emission yield of various substrates

| Material | $\delta_{e,max}$ | $\varepsilon_{\max}$(eV) | $\varepsilon_{e,1}$(eV) | $tan(\theta_{E})$ | $\varepsilon_{r}$ | Reference |
| --- | --- | --- | --- | --- | --- | --- |
| Sapphire | 6.0 | 608 | 52 | 0.34 | 9.3 | [4,5] |
| LiF | 6.0 | 600 | 51 | 0.34 | 9.0 | [6,7] |
| MgF_2_ | 6.1 | 680 | 57 | 0.32 | 5.2 | [8,9] |
| SiO_2_ | 4.35 | 420 | 50 | 0.35 | 3.9 | [10,11] |
| SiC | 2.0 | 400 | 111 | 0.22 | 9.7 | [12,13] |
| GaN | 2.5 | 500 | 108 | 0.23 | 9.8 | [14,15] |
| Diamond | 2.9 | 890 | 163 | 0.19 | 5.9 | [16,17] |

The secondary electron energy spectrum follows a statistical formula as follows [2].

$f\left( \varepsilon_{\mathrm{SE}} \right)=\sum_{i=1}^{2} a_{i}e^{-b_{i}\varepsilon_{\mathrm{SE}}}$

(S-28)

where $\varepsilon_{\mathrm{SE}}$ is the SE’s energy, $a_{i}$ and $b_{i}$ are empirical values. For simplicity, the energy spectrum is averaged over the whole energy range to obtain an average SE energy of $\varepsilon_{e,0}=2.85$eV. According to Equation (S-14), the values of $tan(\theta_{E})$ for the tested substrate materials are listed in Table S-1, demonstrating that $E_{y}=0.2E_{x}\sim0.4E_{x}$.


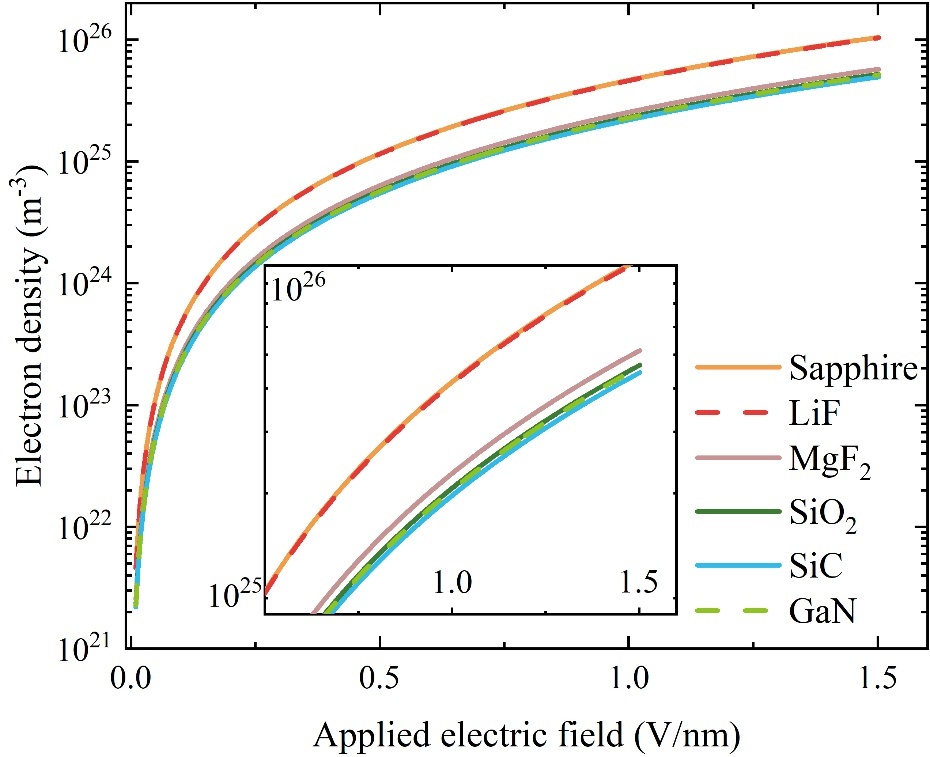


Figure S-5. Calculated SEEA electron density $n_{e,SEEA}$ with increasing $E_{x}$ for tested substrate materials.

Electron density in the SEEA with varying $E_{x}$ is calculated based on Equation (S-19), using the relative permittivity values shown in Table S-1, as shown in Figure S-5. In the $E_{x}$ level of 1 V/nm, the SEEA electron density is in the order of 10^25^ m^-3^.


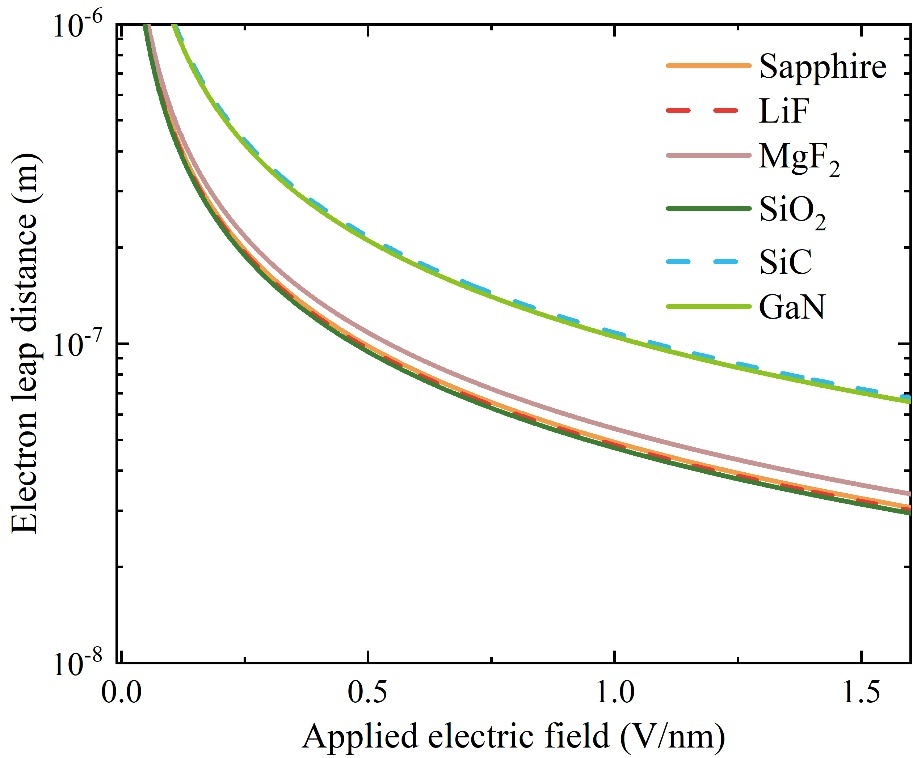


Figure S-6. Calculated electron leap distance $l_{x}$ with increasing $E_{x}$ for tested substrate materials.

The electron leap distance for the tested substrate materials is calculated and is shown in Figure S-6. Its value is ~10 nm for a 1 V/nm $E_{x}$ for all substrate materials, except for SiC and GaN where the leap distance is ~100 nm.

The fraction of SEEA electrons that participate in the ionization avalanche, $f_{\mathrm{ion}}$, depends on the Townsend ionization coefficient, $\alpha_{T}$. For air under atmosphere pressure, it is approximated by:

$\alpha_{T}=A_{T}e^{-B_{T}/E_{x}}$

(S-29)

We take the values of the coefficients $A_{T}$=7.5×10^5^ /m and $B_{T}$=2×10^7^ V/m from experimental measurement[18], where unit conversion is performed. Note that the values of $A_{T}$ and $B_{T}$ are affected by factors including air humidity, temperature, impurity gas, and measurement setup. Combining Equations (S-20) and (S-29), $f_{\mathrm{ion}}$ is calculated and is shown in Figure S-7. Except for SiC and GaN, all substrate materials exhibit $f_{\mathrm{ion}}$ values of less than 5% with $E_{x}$ of 1 V/nm.


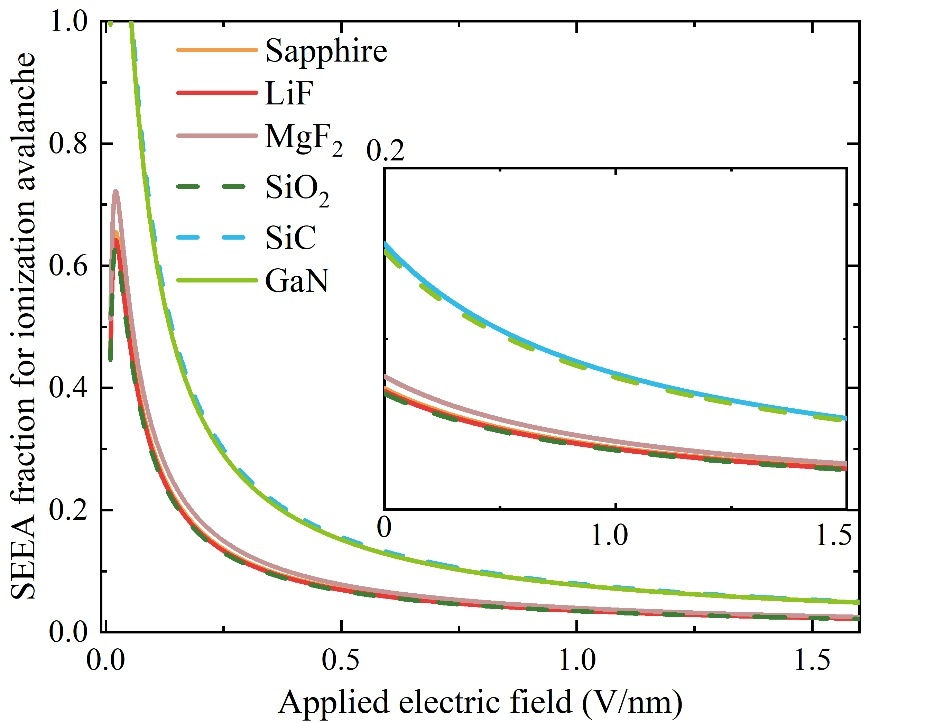


Figure S-7. Calculated SEEA electron fraction for ionization avalanche $f_{\mathrm{ion}}$ with increasing $E_{x}$ for tested substrate materials

The transition time from SEEA to breakdown, $t_{on,2}$, mainly depends on the device gap distance. Numerical values required for the calculation include $L$=200 μm, $D_{e}$=0.5 m^2^/s, $n_{e,crit}$=2×10^24^ m^-3^, $n_{g}$ of atmosphere pressure at 300 K, $\sigma_{\mathrm{mom}}$=5×10^17^ m^-3^.

1. **RF measurement of NPDs with different substrate materials**

Al_2_O_3_ substrate, with THz.


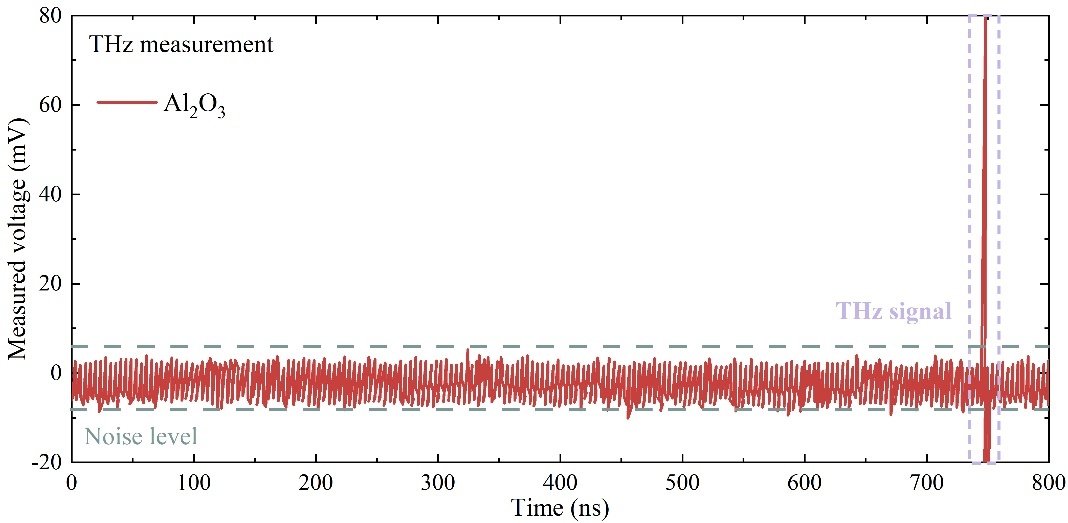


LiF substrate, with THz.


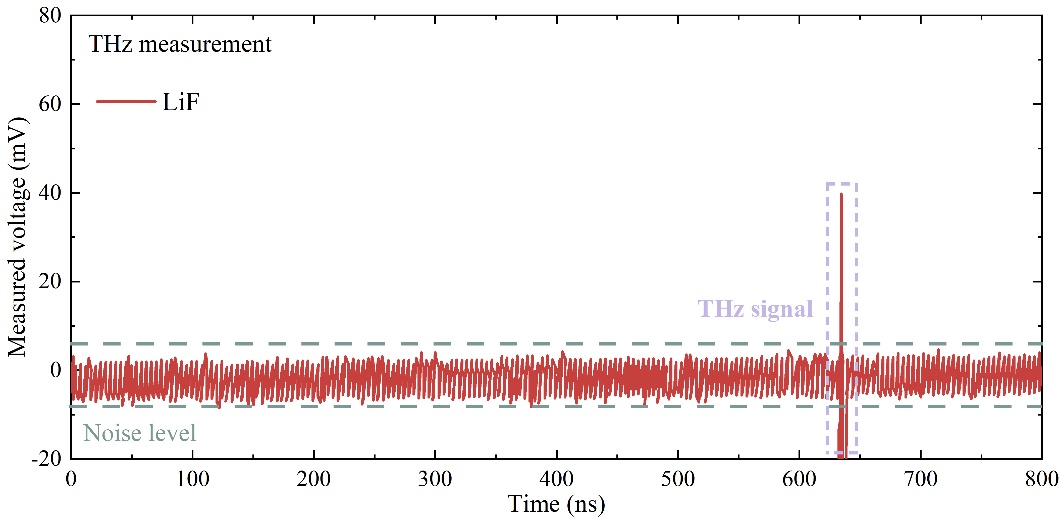


MgF_2_ substrate, with THz.


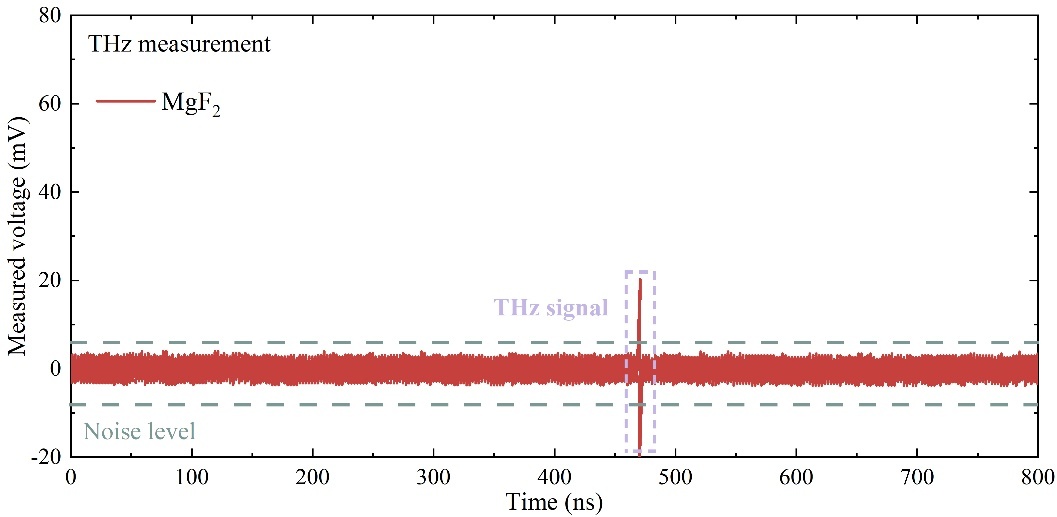


SiO_2_ substrate, with THz.


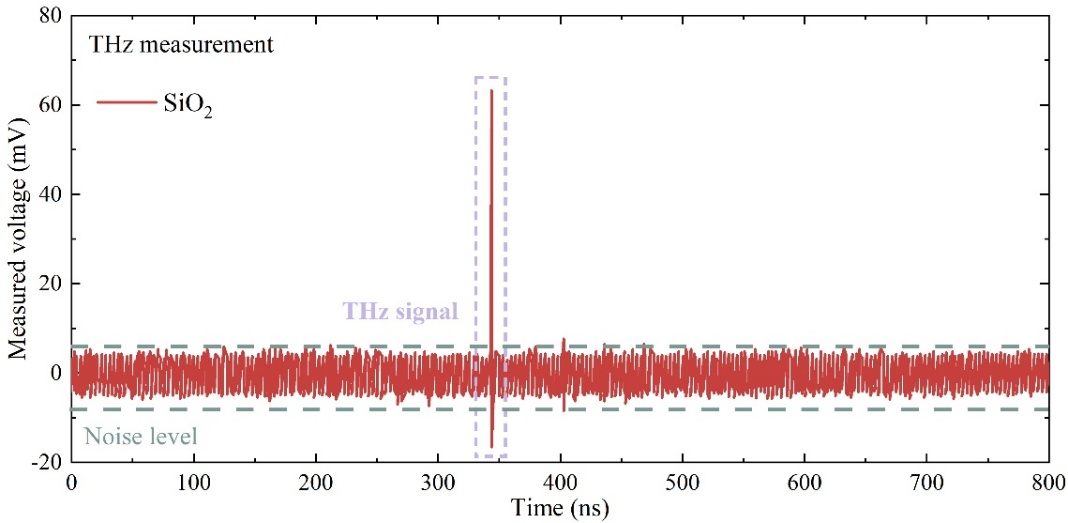


SiC substrate, no THz.


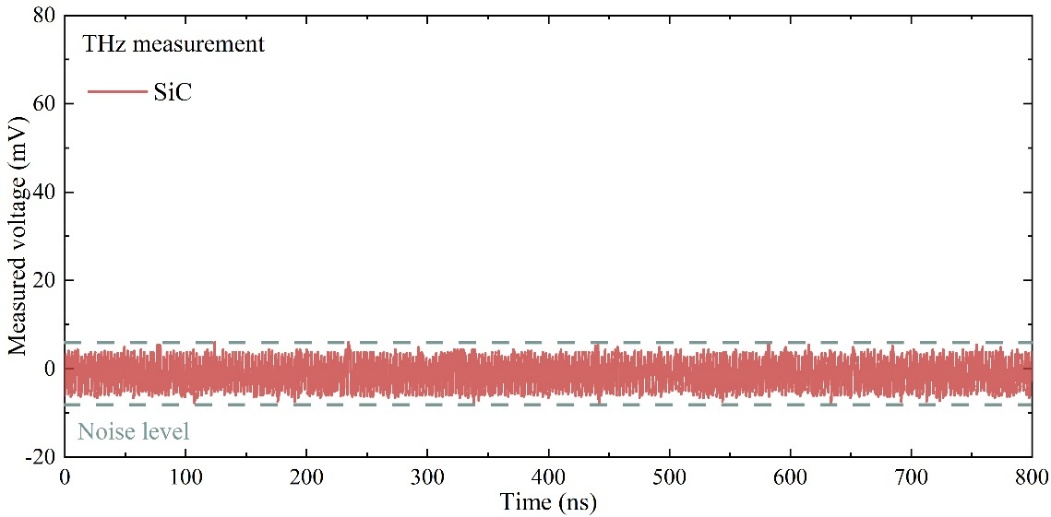


GaN substrate, no THz.


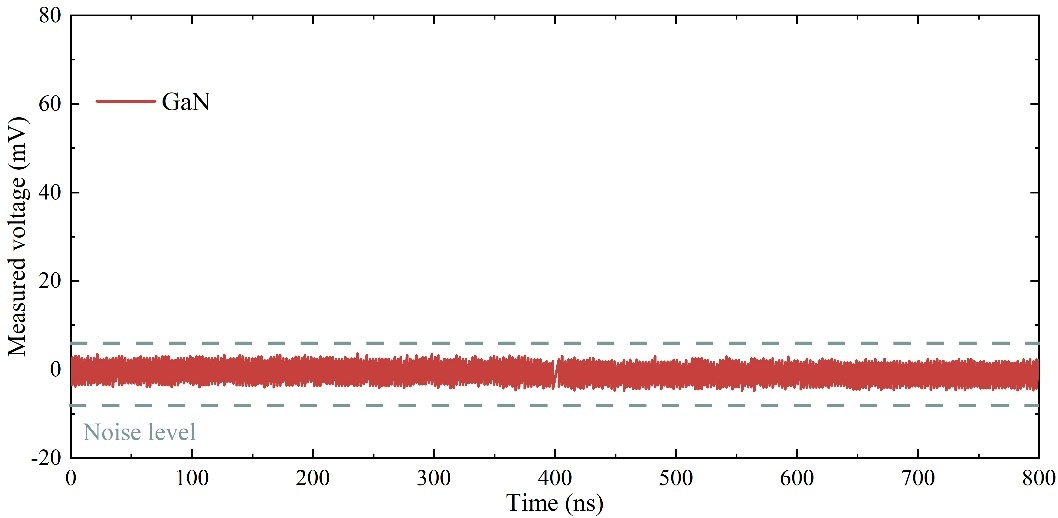


REFERENCES

[1] J. Greenwood, The correct and incorrect generation of a cosine distribution of scattered particles for Monte-Carlo modelling of vacuum systems, Vacuum 67 (2002) 217-222. <https://doi.org/https://doi.org/10.1016/S0042-207X(02)00173-2>.

[2] H.v. Seggern, Charging Dynamics of Dielectrics Irradiated by Low Energy Electrons, IEEE Transactions on Nuclear Science 32 (1985) 1503-1511. <https://doi.org/10.1109/TNS.1985.4333643>.

[3] G.-Y. Sun, Y. Li, S. Zhang, B.-P. Song, H.-B. Mu, B.-H. Guo, A.-B. Sun, G.-J. Zhang, Integrated modeling of plasma-dielectric interaction: kinetic boundary effects, Plasma Sources Science and Technology 28 (2019) 055001. <https://doi.org/10.1088/1361-6595/ab17a3>.

[4] J. Christensen, Electron Yield Measurements of High-Yield, Low-Conductivity Dielectric Materials, Utah State University, 2017.

[5] R.L. Aggarwal, A.K. Ramdas, Physical properties of diamond and sapphire. CRC Press, 2019.

[6] N.R. Whetten, Cleavage in High Vacuums of Alkali Halide Single Crystals—Secondary Electron Emission, Journal of Applied Physics 35 (1964) 3279-3282. <https://doi.org/10.1063/1.1713210>.

[7] C. Andeen, J. Fontanella, D. Schuele, Low-Frequency Dielectric Constant of LiF, NaF, NaCl, NaBr, KCl, and KBr by the Method of Substitution, Physical Review B 2 (1970) 5068-5073. <https://doi.org/10.1103/PhysRevB.2.5068>.

[8] H. Thiemann, R.W. Schunk, Particle-in-cell simulations of sheath formation around biased interconnectors in a low-earth-orbit plasma, Journal of Spacecraft and Rockets 27 (1990) 554-562. <https://doi.org/10.2514/3.26179>.

[9] J. Fontanella, C. Andeen, D. Schuele, Low‐frequency dielectric constants of α‐quartz, sapphire, MgF2, and MgO, Journal of Applied Physics 45 (1974) 2852-2854. <https://doi.org/10.1063/1.1663690>.

[10] L. Huang, Q. Wang, Study on Secondary Electron Yield of Dielectric Materials, Journal of Physics: Conference Series 2433 (2023) 012002. <https://doi.org/10.1088/1742-6596/2433/1/012002>.

[11] R. Singh, R.K. Ulrich, High and Low Dielectric Constant Materials, The Electrochemical Society Interface 8 (1999) 26. <https://doi.org/10.1149/2.F06992IF>.

[12] I.V. Schweigert, A.L. Alexandrov, P.P. Gugin, M.A. Lavrukhin, P.A. Bokhan, D.E. Zakrevsky, Picosecond Breakdown in High-Voltage Open Pulse Discharge With Enhanced Secondary Electron Emission, IEEE Transactions on Plasma Science 45 (2017) 3202-3208. <https://doi.org/10.1109/TPS.2017.2766888>.

[13] L. Patrick, W.J. Choyke, Static Dielectric Constant of SiC, Physical Review B 2 (1970) 2255-2256. <https://doi.org/10.1103/PhysRevB.2.2255>.

[14] J.E. Yater, A. Shih, D.S. Katzer, Secondary Electron Emission Studies of Diamond and GaN Materials, MRS Online Proceedings Library 558 (1999) 551-562. <https://doi.org/10.1557/PROC-558-551>.

[15] L. Cheng, J.-Y. Yang, W. Zheng, Bandgap, Mobility, Dielectric Constant, and Baliga’s Figure of Merit of 4H-SiC, GaN, and β-Ga2O3 from 300 to 620 K, ACS Applied Electronic Materials 4 (2022) 4140-4145. <https://doi.org/10.1021/acsaelm.2c00766>.

[16] A. Shih, J. Yater, P. Pehrsson, J. Butler, C. Hor, R. Abrams, Secondary Electron Emission Studies of Diamond Surfaces, MRS Online Proceedings Library 416 (1995) 461-466. <https://doi.org/10.1557/PROC-416-461>.

[17] A. Itsh’ak, O. Klonsky, Y. Gelbstein, P. Beker, Doping type influence on physical diamond properties, Materials Research Express 9 (2022) 025901. <https://doi.org/10.1088/2053-1591/ac4eb8>.

[18] T. Yalçın, E. Kam, O. Alaçayır, R. Bıyık, Measurement of the first Townsend coefficients in dry air, Radiation Physics and Chemistry 222 (2024) 111876. <https://doi.org/https://doi.org/10.1016/j.radphyschem.2024.111876>.
